# Cost-Effectiveness of Treatments for Musculoskeletal Conditions Offered by Physiotherapists: A Systematic Review of Trial-Based Evaluations

**DOI:** 10.1186/s40798-024-00713-9

**Published:** 2024-04-13

**Authors:** Linda Baumbach, Wiebke Feddern, Benedikt Kretzler, André Hajek, Hans-Helmut König

**Affiliations:** Department of Health Economics and Health Services Research, University Medical Center Hamburg-Eppendorf, Martinistr. 52, 20246 Hamburg, Germany

**Keywords:** Economic evaluation, Physiotherapy, Musculoskeletal condition, Orthopedic

## Abstract

**Background:**

Musculoskeletal conditions are a leading contributor to disability worldwide. The treatment of these conditions accounts for 7% of health care costs in Germany and is often provided by physiotherapists. Yet, an overview of the cost-effectiveness of treatments for musculoskeletal conditions offered by physiotherapists is missing. This review aims to provide an overview of full economic evaluations of interventions for musculoskeletal conditions offered by physiotherapists.

**Methods:**

We systematically searched for publications in Medline, EconLit, and NHS-EED. Title and abstracts, followed by full texts were screened independently by two authors. We included trial-based full economic evaluations of physiotherapeutic interventions for patients with musculoskeletal conditions and allowed any control group. We extracted participants' information, the setting, the intervention, and details on the economic analyses. We evaluated the quality of the included articles with the Consensus on Health Economic Criteria checklist.

**Results:**

We identified 5141 eligible publications and included 83 articles. The articles were based on 78 clinical trials. They addressed conditions of the spine (n = 39), the upper limb (n = 8), the lower limb (n = 30), and some other conditions (n = 6). The most investigated conditions were low back pain (n = 25) and knee and hip osteoarthritis (n = 16). The articles involved 69 comparisons between physiotherapeutic interventions (in which we defined primary interventions) and 81 comparisons in which only one intervention was offered by a physiotherapist. Physiotherapeutic interventions compared to those provided by other health professionals were cheaper and more effective in 43% (18/42) of the comparisons. Ten percent (4/42) of the interventions were dominated. The overall quality of the articles was high. However, the description of delivered interventions varied widely and often lacked details. This limited fair treatment comparisons.

**Conclusions:**

High-quality evidence was found for physiotherapeutic interventions to be cost-effective, but the result depends on the patient group, intervention, and control arm. Treatments of knee and back conditions were primarily investigated, highlighting a need for physiotherapeutic cost-effectiveness analyses of less often investigated joints and conditions. The documentation of provided interventions needs improvement to enable clinicians and stakeholders to fairly compare interventions and ultimately adopt cost-effective treatments.

**Supplementary Information:**

The online version contains supplementary material available at 10.1186/s40798-024-00713-9.

## Key Points

- Several high-quality economic evaluations of physiotherapeutic treatments for the back and knee exist
- Economic evaluations of other joints are rare
- Physiotherapeutic interventions are often cost-effective over treatments provided by other health professionals
- The description of provided interventions in cost-effectiveness analyses needs improvement, to allow fair treatment comparisons

**Supplementary Information:**

The online version contains supplementary material available at 10.1186/s40798-024-00713-9.

## Background

### Rationale

Globally about 1.71 billion people suffer from a musculoskeletal condition [1]. Most adults in rehabilitation within Germany present with this diagnosis [2]. These patients have an increased risk of developing additional chronic diseases and mental health problems, which further increase the patients’ burden [3, 4]. Rehabilitation programs, aiming at reducing these patients' burden and their duration of sick leave, are often planned, and implemented by physiotherapists. Their and other therapeutic services account for 7% of the health expenditures in Germany (2021) and thus cause a financial burden for society [5].

Physiotherapeutic treatments have been used before clinical studies were conducted, but in the last decades, several interventions for specific diseases have been evaluated regarding their clinical effectiveness—although some intervention-disease-combinations remain yet unexplored. In recent years, besides their clinical effectiveness, the costs involved with a treatment have become additionally important to the stakeholders. The combined information supports their decision on whether a treatment should be implemented or de-implemented [6]. Full economic evaluations furnish this information on the anticipated costs along with the expected clinical outcomes of an intervention, enabling comparisons between various disease interventions and by this providing insight into possible cost savings. The costs presented in such analyses consider either healthcare costs alone or both healthcare costs and societal costs [6].

Several studies have already demonstrated that physiotherapists offer treatments worth the money [7, 8]. One review provides an overview of economic evaluations for treatments of neurological conditions offered by physiotherapists [9] and another review evaluates the cost-effectiveness of physical exercise, which is one of the treatment modalities offered by physiotherapists, for various health conditions [10]. However, an overview of existing economic evaluations of different physiotherapeutic treatment modalities for patients with musculoskeletal conditions is missing. Such an overview allows physiotherapists to easily identify relevant publications. Furthermore, it enables policymakers to easily identify relevant studies and researchers to plan future investigations.

### Objectives

In this review, we therefore aim to:

- provide an overview of existing full economic evaluations of interventions for patients with musculoskeletal conditions offered by physiotherapists.
- shed light on the cost-effectiveness of physiotherapeutic interventions for specific musculoskeletal health conditions.
- highlight for which health conditions further research is needed.

## Methods

### Protocol and Registration

A protocol for this review was published and additionally registered at Prospero (CRD42021276050) [11]. We followed our protocol but decided to report the findings of the identified trial and model-based economic evaluations separately. This allows us to fully account for and address the heterogeneous aspects of the two study types. Here we present our findings from the trial-based publications. We report our results following the PRISMA statement.

### Eligibility Criteria

We included full economic evaluations of physiotherapeutic interventions for patients with musculoskeletal conditions. To specify our inclusion and exclusion criteria, we used the PICOS acronym (population, intervention, control group, outcome, study type).

Our population of interest suffered from a musculoskeletal condition. If the majority had another primary disease or if the participants had intellectual disabilities, we excluded the publication.

We included publications where physiotherapists provided one of the intervention/control group treatments alone. We excluded publications if the treatment of interest was offered by an interdisciplinary team, non-healthcare professionals, or mostly by a different profession. If the physiotherapeutic treatment of interest was combined with another treatment, this needed to be provided in a comparator group as well. Thus, an isolated incremental effect evaluation of a physiotherapeutic intervention needed to be possible. Economic evaluations of E-intervention were excluded.

We allowed any type of control group including wait-and-see, usual care, placebo, and alternative treatments, and excluded publications where no control group was considered.

Our outcome needed to be the result of a full health economic evaluation, including cost-effectiveness ratios, and cost-utility ratios.

Economic evaluations based on models and clinical trials were included during the screening process. We excluded Delete 'study types like' conference abstracts, reviews, books, and articles with no access and studies without results, e.g. protocols. In this publication, we additionally excluded model-based studies during the full-text screening, to reduce heterogeneity of included studies and allow a fair comparison of the study quality.

Finally, the economic evaluation needed to be published in Danish, English, or German.

### Information Sources

We searched for relevant publications in the databases Medline (through PubMed), EconLit, and NHS-EED (can only be searched up to March 2015). The initial search was performed at the end of January 2022 and the final update was performed on the 8th of December 2023.

### Search Strategy

We used the three main search terms ‘economic evaluation’, ‘physiotherapy’, and ‘orthopedic’ to develop a search matrix. For each of the main search terms we collected synonyms and combined them with an ‘OR’ in a search. Afterwards, we merged the three synonym searches with two ‘AND’. Utilizing asterisks reduced the number of search terms but allowed identifying relevant articles. Our search string in PubMed consequently was: ((((economic analy*) OR (cost analy*) OR (cost benefit*) OR (cost utility) OR (cost effectiveness) OR (economic evaluation))) AND (((orthopaedic rehabilitation) OR (physical rehabilitation) OR (exercise therap*) OR (conservative therap*) OR (conservative management) OR (conservative treatment) OR (exercise training) OR (physiotherap*))) AND (((muskuloskeletal) OR (chirur*) OR (orthop*) OR (osteoarthritis) OR (back pain)))). "Details on the PubMed search" are also available in Additional file 1: Table S1.

### Selection Process

After removing duplicates, we applied a two-step study selection process. In the first step, we screened the title and abstracts for our inclusion and exclusion criteria. In the second step, we evaluated the full texts. Both steps were conducted by two independent reviewers at any time (LB, WF, BK). After each step, the involved authors compared their results and resolved disagreements via discussions or consulting a third reviewer (HHK) if needed.

### Data Collection Process

The spreadsheet for data extraction was independently tested on three included publications by two authors (LB, BK). The authors discussed uncertainties, adjusted the labeling in the spreadsheet, and repeated the process. The authors agreed in the second round and LB proceeded with the data extraction. WF became involved in the data extraction process after she compared the data extraction results of three publications with LB, and no disagreement occurred.

### Data Items

In total, we extracted the following items per publication: authors, year of publication, sample size, mean age, the proportion of female sex, health condition, location, type of economic analysis, cost perspective, economic effect measure, time horizon, study design, setting, and frequency, intensity, duration as well as the type of intervention, further control intervention, cost-effectiveness results, and finally the author’s conclusion. If the modalities of an intervention were not presented in a publication, but a reference was mentioned, we extracted the information from the cited publication. Further, in articles with several physiotherapeutic intervention or control groups, we ordered the interventions of interest as primary, secondary and so on. We prioritized basic interventions as primary, meaning interventions which could be most easily provided by physiotherapists—without further training.

### Critical Appraisal of the Included Publications

We utilized the Consensus on Health Economic Criteria checklist for evaluating the quality of the included articles [12]. This tool consists of 19 questions which can be answered with ‘yes’ or ‘no’ each. Each ‘yes’ indicates good quality, whereas a ‘no’ indicates limited quality.

Two authors (LB and HHK) independently assessed the quality of three articles. After discussing uncertainties LB proceeded with the evaluation. WF got involved in the assessment after she and LB agreed on the quality of three, independently evaluated, articles.

### Synthesis of Results

We provide a descriptive overview of the available literature. We categorized the economic evaluations based on the affected body parts. Afterwards, we grouped all cost-effectiveness comparisons, which are partly between two physiotherapeutic interventions, and partly between a physiotherapeutic and another intervention, of the included articles according to the four quadrants of a cost-effectiveness plane (I. more costly, more effective; II. more costly, less effective (dominated); III. less costly, less effective; IV. less costly, more effective (dominant)). The IV. quadrant is always assumed to be cost-effective, and the II. quadrant is always dominated, hence not cost-effective in the performed comparison. However, whether the interventions are cost-effective in the other two quadrants (I. and III.), depends on the stakeholder’s willingness to pay for an extra benefit in a health outcome. As an example, if a health system is not willing to pay the additional costs for the gain in health outcomes in the comparison group, our (primary) interventions in the III. quadrant would be cost-effective. However, if the healthcare system is willing to pay the additional costs to achieve the additional benefit in the health outcome, our (primary) interventions grouped in the I. quadrant would be cost-effective. Thus, to determine if interventions of the I. and III. quadrants are cost-effective, we would need to know the amount of money the healthcare system or society is willing to pay for a one-unit gain in the respected health outcome. However, this is beyond the scope of this review. For comparisons between two physiotherapeutic interventions, we defined one intervention as the primary intervention of interest. These primary physiotherapeutic interventions were more likely offered solely by physiotherapists and required least additional training/courses for the physiotherapists.

## Results

### Selection of Publications

**Fig. 1:**
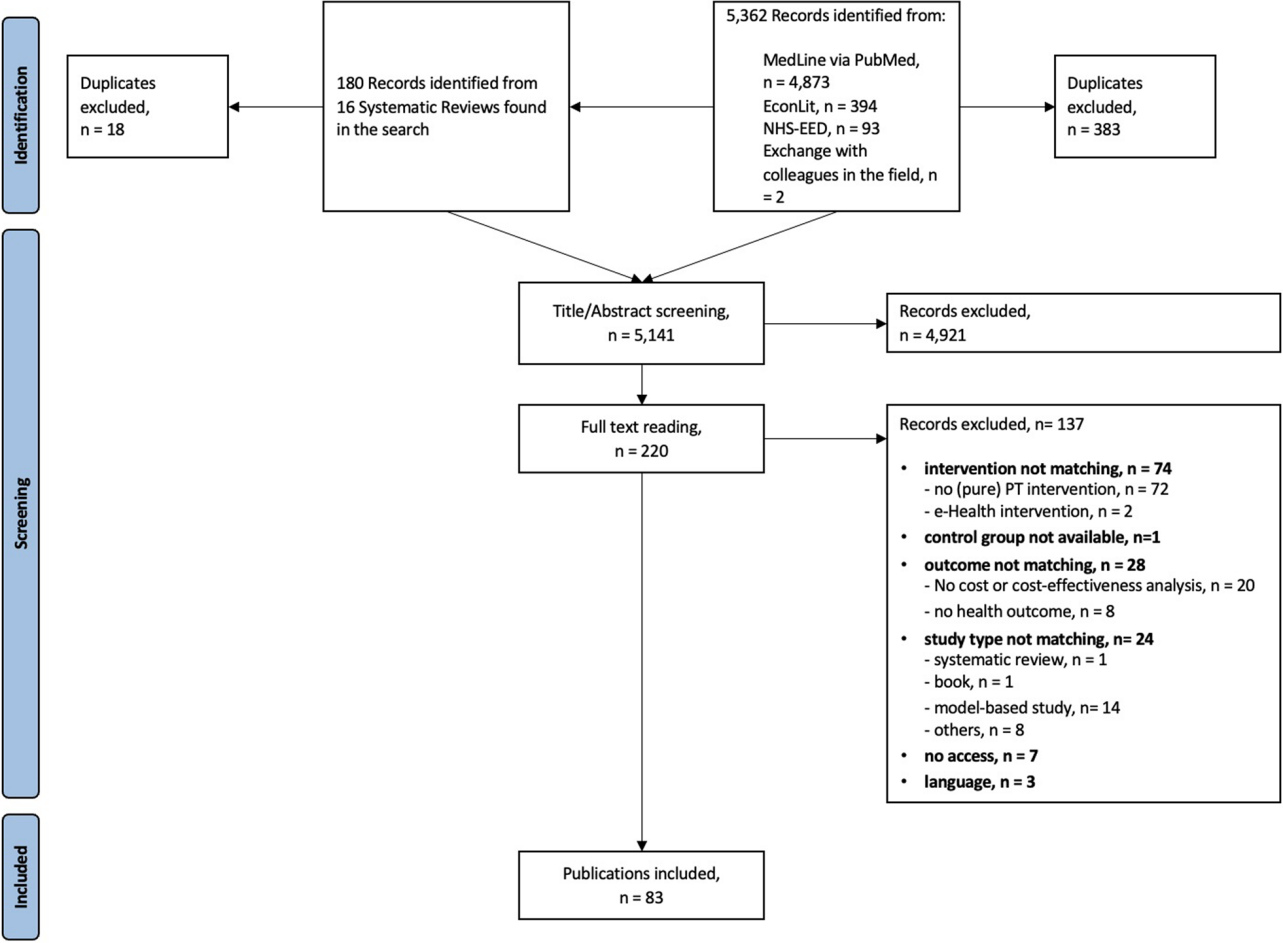
Flow chart of the study selection process

Our search findings and the study selection process are visualized in the flowchart Fig. 1. In total, we identified 5141 eligible publications of which 83 met our inclusion criteria. A list of the publications excluded during the full-text screening process including an indication of the reason for exclusion is provided in Additional file 2: Table S2.

### Characteristics of the Economic Evaluations

**Fig. 2:**
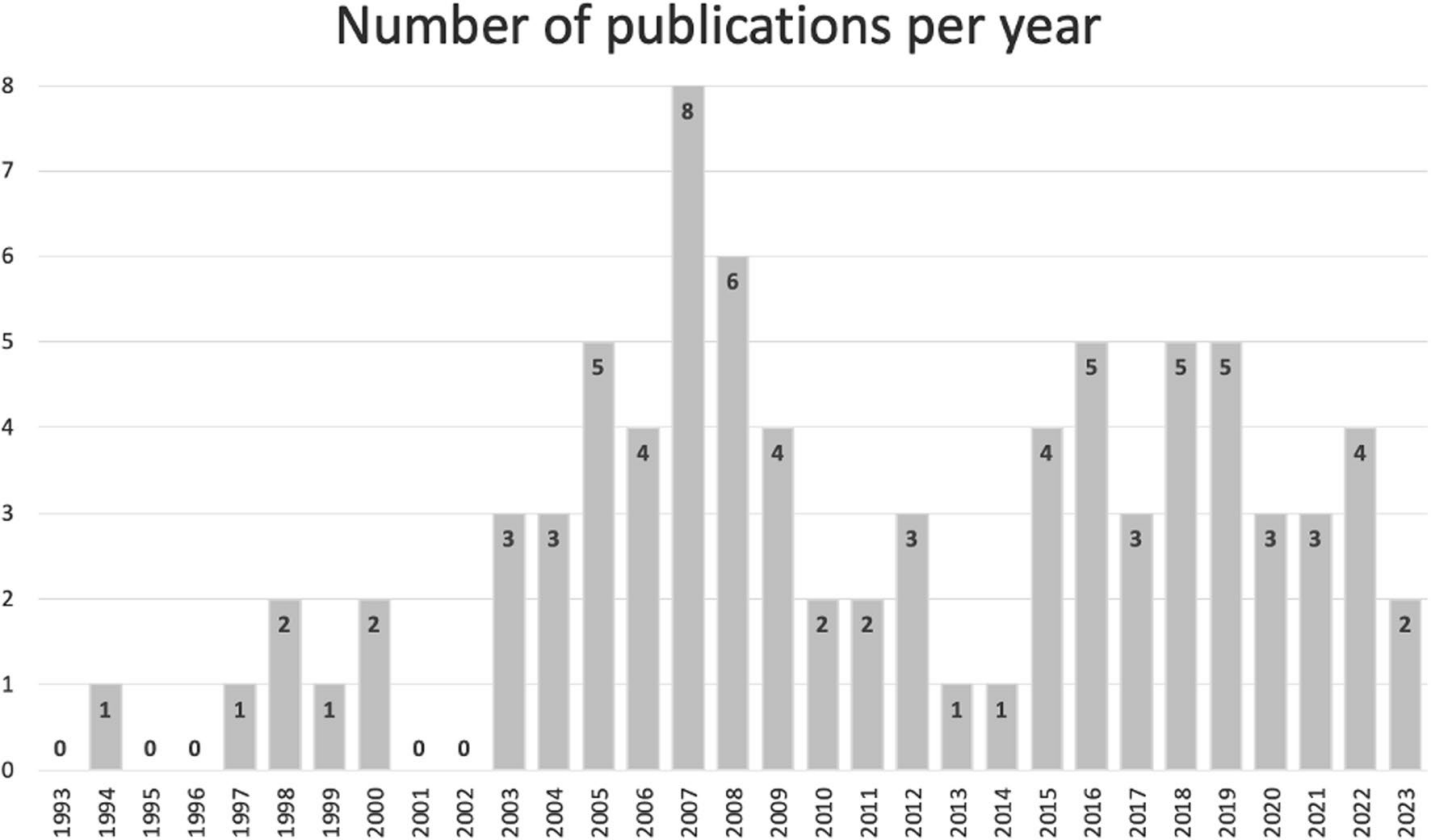
Years of publication of the included publications

**Fig. 3:**
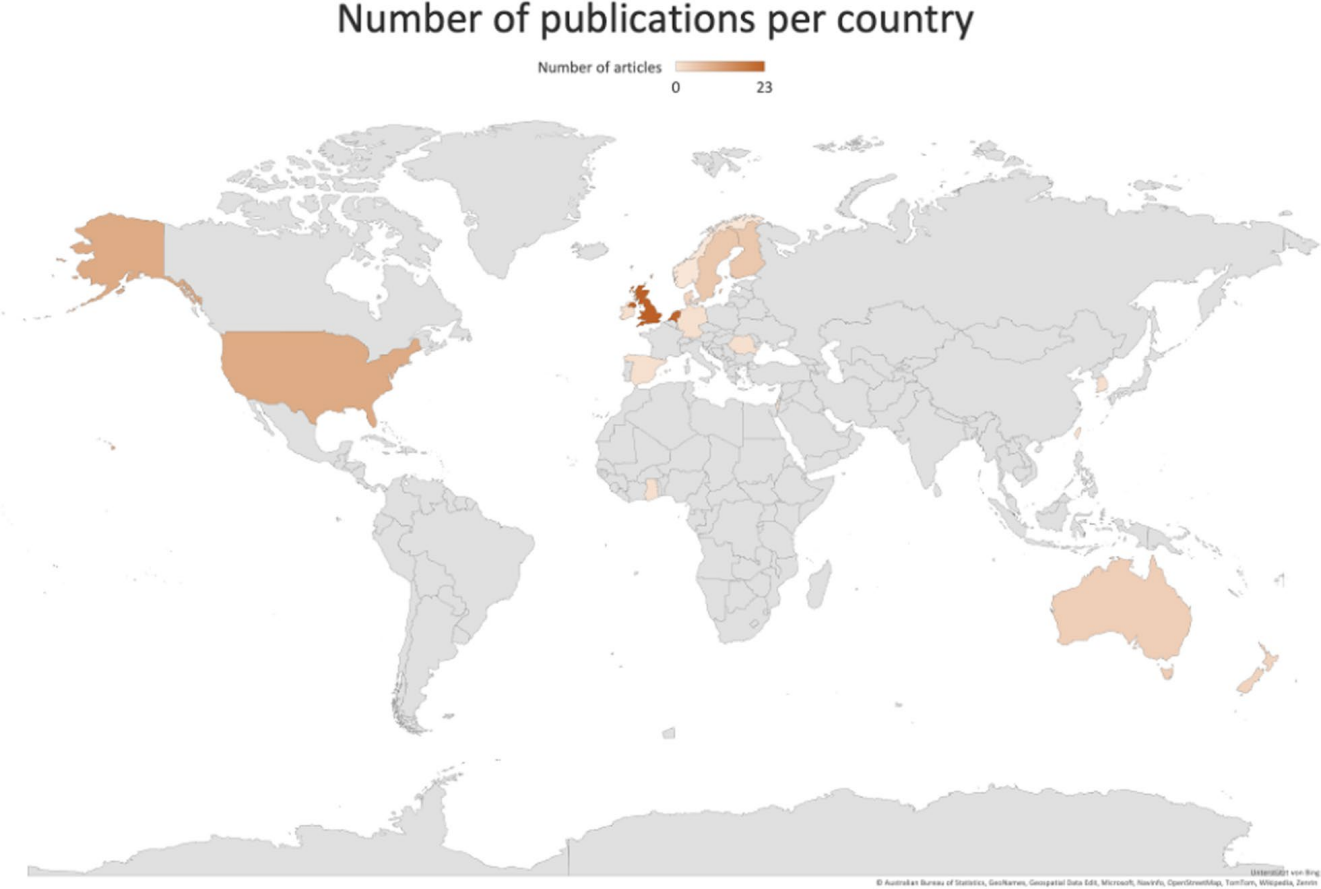
Overview of the origin of included publications

Table 1 summarizes our included 83 articles which were based on 78 clinical trials [13–95]. Niemistö et al., Barker et al., Skargren et al. as well as Hurley et al. published two articles each building on one trial [17, 18, 51, 52, 72, 81, 82, 96]. Abbott et al. and Pinto et al. utilized data from the MOA RCT [13, 74]. All articles were published between 1994 and 2023 (Fig. 2). The majority of the 78 clinical trials were RCTs (n = 71) and 71 of the articles originated from the Western world. The UK (n = 23) and the Netherlands (n = 22) contributed more than 50% of the articles (Fig. 3). The time horizon of the economic evaluations varied between 5 days and 36 months. In Table 1 the included articles are grouped by their addressed body parts: spine (n = 39), upper limb (n = 8), lower limb (n = 30), and other conditions (n = 6). The most frequently investigated conditions were low back pain (n = 25) and hip and knee osteoarthritis (n = 16). In most included samples the mean age was between 45 and 65 and the distribution of female and male participants was between 40 to 60%. If a sample deviated from this, the mean age and female percentage were added in Table 1.

**Table 1:** Overview of the included trial-based economic evaluations

Study ID	Patients' characteristics	Country	Study design, type of economic analysis	Cost perspective	Effect measure	Time horizon
Spine						
*Back*						
A Barker et al. 2019 [17] B Barker et al. 2020 [18]	n = 615 Mean age: 72 Female: 86% Osteoporotic vertebral fracture	UK	RCT (PROVE-trial) Cost-utility	Healthcare perspective, Societal perspective	QALY (EQ-5D)	12 months
Müller et al. 2019 [71]	n = 2324 patients Back pain	DE	Prospective cohort study Cost-effectiveness	Healthcare perspective, sick leave	Pain intensity (Graded chronic back pain status)	24 months
Søgaard et al. 2008 [84]	n = 90 lumbar spinal fusion	DK~	RCT Cost-effectiveness	Societal perspective	Pain- and disability index scales of the low back pain rating scale	24 months
*Low back pain*						
Aboagye et al. 2015 [14]	n = 159 female: (i1) 72% (i2) 62% (c) 80% LBP	SE	RCT Cost-effectiveness	Societal perspective	QALY (EQ -5D)	12 months
Ankjær-Jensen et al. 1994 [15]	n = 172 Mean age: 44 LBP (herniated disc)	DK	Retrospective cohort study Cost-effectiveness	Societal perspective	Low back pain rating scale	(i) 12 months c) 22 months
Apeldoorn et al. 2012 [16]	n = 156 Mean age: (i) 43 (c) 42 LBP (chronic)	NL	RCT cost-effectiveness	Societal perspective	QALY (EQ-5D)	12 months
Bello et al. 2015 [21]	n = 62 Mean age: (i) 43 (c) 45 LBP (chronic)	GH	Feasibility intervention NA	Healthcare perspective	SF-36, numeric rating scale	3 months
Burton et al. 2004 [26]	n = 1287 LBP (non-specific)	UK	RCT Cost-utility, cost-effectiveness	Healthcare perspective	QALY (EQ -5D)	12 months
Canaway et al. 2018 [27]	n = 220 Mean age: 42 LBP	IL	Prospective cohort study Cost-effectiveness	Healthcare perspective	QALY (SF-12)	12 months
Carr et al. 2005 [28]	n = 237 Mean age: (i) 42 (c) 43 LBP	UK	RCT Cost-effectiveness	Healthcare perspective	RMDQ	12 months
Cherkin et al. 1998 [29]	n = 321, Mean age: 41 LBP (chronic) [12+ weeks]	US	RCT Cost-effectiveness	Healthcare perspective	Bothersomeness of symptoms, RDS	Short-term: 3 months Long-term: 12–24 months
Critchley et al. 2007 [32]	n = 150 Mean age: 44 Female: (i1) 71% (i2) 62% (c) 69% LBP (acute) [symptoms < 90 days]	UK	RCT Cost-effectiveness	Healthcare perspective	QALY (EQ-5D)	18 months
Fritz et al. 2008 [38]	n = 471 Mean age: 41 LBP (acute) [without clinical signs of nerve root, symptoms < 16 days]	US	Case–control Cost-effectiveness	Healthcare perspective	OSW, pain rating	24 months
Fritz et al. 2017 [39]	n = 220 Mean age: (i) 38 (c) 37 LBP [symptoms for 6 weeks to 6 months]	US	RCT Cost-effectiveness	Societal perspective	QALY (EQ-5D)	12 months
Hahne et al. 2017 [43]	n = 300 Mean age: (i) 43 (c) 46 LBP (chronic) [symptoms 6+ weeks]	AU	RCT Cost-utility	Healthcare perspective	QALY (EQ-5D)	12 months
Herman et al. 2008 [45]	n = 75 LBP [symptoms for 4+ weeks]	US	RCT Cost-effectiveness	Main: societal perspective; additional: employer, participant	QALY (SF-6D)	6 months
Hlobil et al. 2007 [46]	n = 134 [sick-listed worker] mean age: (i) 39 (c) 37 LBP (chronic)	NL	RCT Cost–benefit	Societal perspective	Lost productivity days	36 months
Hurley et al. 2015 [50]	n = 246 LBP [symptoms 3+ months]	IE	RCT Cost-utility	Healthcare perspective	QALY (EQ-5D)	12 months
Johnson et al. 2007 [55]	n = 234 LBP	UK	RCT Cost-utility	Healthcare perspective	QALY (EQ-5D)	12 months
Karjalainen et al. 2003 [57]	n = 164 mean age: (i1) 44 (i2) 44 (c) 43 [25–61 y.] LBP	FI	RCT Cost–benefit	Healthcare perspective	Bothersomeness and frequency of pain, daily symptoms, generic health-related quality of life, intensity of pain, ODI, sick leave,	12 months
Kim et al. 2020 [59]	n = 56 [BMI 17–30] Mean age: (i) 48 (c) 39 Female: (i) 25% (c) 29% LBP (chronic)	KR	RCT Cost-effectiveness	Healthcare perspective	Functional rating index, Multidimensional Personality Questionnaire, VAS	3 weeks
A Niemistö et al. 2003 [72] B Niemistö et al. 2005 [73]	n = 204 Mean age: (i) 37 (c) 37 [24–46] LBP (subacute and chronic) [symptoms 6+ weeks]	FI	RCT Cost-effectiveness	Societal perspective	A VAS B ODI, VAS	A 12 months B 24 months
Rivero-Arias et al. 2006 [77]	n = 286 mean age: (i) 42 (c) 40 LBP (chronic) [symptoms 3+ months]	UK	RCT Cost-utility	Healthcare perspective, societal perspective	QALY (EQ-5D)	12 months
Smeets et al. 2009 [83]	n = 160 Mean age: (i1) 43 (i2) 42 (c) 43 LBP	NL	RCT Cost-effectiveness, cost-utility	Societal perspective	RMDQ, QALY (EQ-5D)	12 months
Suni et al. 2018 [87]	n = 219 [health professionals] Female: 100% LBP (chronic)	FI	RCT Cost-effectiveness	Healthcare perspective, sick leave	QALY (SF-6D)	12 months
van der Roer et al. 2008 [93]	n = 114 [have a health insurance with company AGIS] LBP [symptoms < 12 weeks]	NL	RCT Cost-effectiveness	Societal perspective	General perceived effect (6-point-scale), pain-rating-scale, EQ-5D, RMDQ	12 months
Whitehurst et al. 2007 [95]	n = 299 LBP (chronic)	UK	RCT cost-effectiveness, cost-utility	Healthcare perspective	RMDQ, QALY (EQ-5D)	12 months
*Neck*						
Bosmans et al. 2011 [24]	n = 146 Neck pain (subacute)	NL	RCT Cost-effectiveness, cost-utility	Societal perspective	Patient perceived recovery, QALY (SF-6D)	12 months
Korthals-de Bos et al. 2003 [62]	n = 183 Neck pain	NL~	RCT Cost-effectiveness, cost-utility	Societal perspective	EQ, functional disability, pain intensity, patient perceived recovery	12 months
Leininger et al. 2016 [63]	n = 241 Mean age: 73 Neck pain (chronic) [symptoms 3+ months]	US	RCT Cost-effectiveness	Societal perspective	QALY (SF-6D)	12 months
Lewis et al. 2007 [64]	n = 350 Female: 63% (in total) Neck disorders (non-specific)	UK	RCT Cost-effectiveness, cost-utility	Healthcare perspective, societal perspective	Northwick Park Questionnaire, QALY (EQ-5D)	6 months
Manca et al. 2006 [68]	n = 268 Female: (i) 62% (c) 66% Neck pain [musculoskeletal origin, symptoms 2+ weeks]	UK	RCT Cost-effectiveness	Healthcare perspective	QALY (EQ-5D)	3 months, 12 months
Van Dongen et al. 2016 [94]	n = 181 Female: (i) 62% (c) 62% neck pain (subacute and chronic)	NL	RCT cost-effectiveness, cost-utility	Societal perspective	Neck Disability Index—Dutch Version, patient's perceived recovery	12 months
*Others/mixed*						
Denninger et al. 2018 [34]	n = 447 Female: 72% Back pain or neck pain	US	Retrospective cohort Cost-effectiveness	Healthcare perspective	EQ-5D, NPRS, Oswestry Disability Index/Neck Disability Index, Patient Health Questionnaire-4	24 months
Manca et al. 2007 [67]	n = 315 Back pain or neck pain [non-systematic origin, symptoms 2+ weeks]	UK	RCT Cost-effectiveness	Healthcare perspective	QALY (EQ-5D)	12 months
A Skargren et al. 1997 [82] B Skargren et al. 1998 [81]	n = 323 Mean age: (i) 41 c) 41 back or neck pain	SE	RCT Cost–benefit	Healthcare perspective	General Health (scale), ODS, VAS	A 6 months B 12 months
*Upper limb*						
Bergman et al. 2010 [23]	n = 142 Shoulder complaints	NL	RCT Cost-effectiveness	Societal perspective	Patient perceived recovery	6 months
Commbes et al. 2016 [30]	n = 154 Female: (i1) 36 (i2) 39 (c1) 38 (c2) 38 epicondylitis lateralis [> 6 weeks duration]	AU	RCT Cost-utility	Societal perspective	QALY (EQ-5D)	12 months
Fernandez-de-Las-penjas et al. 2019 [37]	n = 120 Female: 100% Carpal tunnel syndrom	ES	RCT Cost-effectiveness	Societal perspective	QALY (EQ-5D)	12 months
Geraets et al. 2006 [41]	n = 176 Shoulder complaints (chronic)	NL	RCT Cost–benefit	Societal perspective	EQ-5D, main complaints, Shoulder Disability Questionnaire	12 months
Hopewell et al. 2021 [48]	n = 708 Rotator cuff disease	UK	RCT Cost-utility	Healthcare perspective	QALY (EQ-5D)	12 months
James et al. 2005 [53]	n = 207 Shoulder pain [new episode]	UK	RCT Cost-consequences	Healthcare perspective	Disability score, EQ-5D	6 months
Korthals-de Bos et al. 2004 [61]	n = 183 Epicondylitis lateralis	NL	RCT Cost-effectiveness, cost-utility	Societal perspective	Cost effectiveness: general improvement, pain during the day, PFFQ cost-utility: EQ	12 months
Struijs et al. 2006 [86]	n = 180 Epicondylitis lateralis [symptoms 6+ weeks]	NL	RCT Cost-effectiveness, cost-utility	Societal perspective	EQ, pain-free function questionnaire, pain most serious complaint, severity of complaint, success rate	12 months
*Lower limb*						
*Hip*						
Fusco et al. 2019 [40]	n = 80 Female: 0% Hip replacement	UK	RCT Cost-effectiveness, cost-utility	Healthcare perspective	QALY (EQ-5D)	12 months
Griffin et al. 2022 [42]	n = 358 Mean age: (i) 35 (c) 35 Female: (i) 42% (c) 36% femoroacetabular impingement syndrome	UK	RCT (UK FASHioN RCT) Cost-effectiveness	Healthcare perspective, societal perspective	QALY (EQ-5D)	12 months
Juhakoski et al. 2011 [56]	n = 118 Mean age: (i) 67 (c) 66 Female: (i) 68% (c) 72% Hip osteoarthritis	FI	RCT Cost-effectiveness	Healthcare perspective	SF-36, WOMAC	24 months
Tan et al. 2016 [88]	n = 203 Mean age: (i) 65 (c) 67 Female: (i) 62% (c) 55% Hip osteoarthritis	NL	RCT Cost-utility	Healthcare perspective, societal perspective	QALY (EQ-5D)	12 months
*Knee*						
Barton et al. 2009 [20]	n = 389 Female: 66% (in total) Knee pain [BMI > = 28; age = 45+]	UK	RCT Cost-effectiveness	Healthcare perspective	QALY (EQ-5D)	24 months
Bennell et al. 2016 [22]	n = 222 [50+] Knee osteoarthritis	AU	RCT Cost-effectiveness	Healthcare perspective	QALY (EQ-5D)	12 months
Eggerding et al. 2021 [35]	n = 167 Mean age: (i) 31 (c) 31 ACL tear [recent ACL tear, max. 2 month ago]	NL+	RCT Cost-utility	Healthcare perspective, Societal perspective	QALY (EQ-5D)	24 months
Ho-Henriksson et al. 2022 [47]	n = 69 Female: (i) 60% (c) 68% Knee osteoarthritis	SE	RCT Cost-effectiveness	Health care perspective, societal perspective	QALY (EQ-5D)	12 months
Huang et al. 2012 [49]	n = 243 Mean age: (i) 70 (c) 71 Female: (i) 70% (c) 74% Total knee replacement [unilateral TKA, due to OA]	TW	RCT Cost-effectiveness	Healthcare perspective	Knee ROM, Length of stay, VAS	5 days
A Hurley et al. 2007 [52] B Hurley et al. 2012 [51]	n = 418 Mean age: (i1) 66 (i2) 68 (c) 67 [50+] female: (i1) 25% (i2) 22% (c) 23% Knee pain (chronic) [symptoms 6+ months]	UK	A RCT (ESCAPE-Knee-Study) Cost-effectiveness, cost-utility B RCT (ESCAPE-Knee-Study) Cost-effectiveness	Healthcare perspective, social care payer perspective	A WOMAC, QALY (EQ-5D) B WOMAC	A 6 months B 30 months
Jessep et al. 2009 [54]	n = 64 Mean age: (i) 66 (c) 67 [> 50] Female: (i) 63% (c) 76% Knee pain (chronic)	UK	RCT Cost–benefit	Healthcare perspective	QALY (EQ-5D)	12 months
Kigozi et al. 2018 [58]	n = 514 Knee osteoarthritis	UK	RCT (BEEP-trial) Cost-effectiveness, cost-utility	Healthcare perspective	QALY (EQ-5D)	18 months
Knoop et al. 2023 [60]	n = 328 Mean age: (i) 66 (c) 64 [40–85] Female: (i) 63% (c) 64% Knee osteoarthritis	NL	RCT Cost-utility	Societal perspective	QALY (EQ-5D)	12 months
McCarthy et al. 2004 [69]	n = 214 Knee osteoarthritis	UK	RCT (GRASP-RCT) cost-effectiveness	Healthcare perspective	QALY (EQ-5D)	12 months
Mitchell et al. 2005 [70]	n = 114 Mean age: (i) 70 (c) 71 Total knee replacement	UK	RCT Cost-effectiveness	Healthcare perspective	SF-36, WOMAC	15 months
Pryymachenko et al. 2021 [75]	n = 75 Female: (i1) 63% (i2) 67% (i3) 63% (c) 58% Knee osteoarthritis	NZ	RCT (MOA2-Trial) Cost-effectiveness	Healthcare perspective, societal perspective	QALY (EQ-5D)	24 months
Rhon et al. 2022 [76]	n = 156 Female: (i) 37% (c) 38% Knee osteoarthritis	US	RCT Cost-effectiveness	Healthcare perspective	QALY (EQ-5D)	12 months
Sevick et al. 2000 (ex) [78]	n = 439 Mean age: (i1) 69 (i2) 68 (c) 69 [60+] Female: (i1) 69% (i2) 73% (c) 69% Knee osteoarthritis	US	RCT Cost-effectiveness	Healthcare perspective	Car task, lifting and carrying task, Self-reported disability score, stair climb, 6-min walking distance	18 months
Sevick et al. 2009 [80]	n = 316 Mean age: (i1) 68 (i2) 69 (i3) 69 (c) 69 Female: (i1) 72% (i2) 74% (i3) 74% (c) 68% Knee osteoarthritis	US~	RCT (ADAPT-trial) Cost-effectiveness	Payer perspective	Stair climb, weight, WOMAC function, WOMAC pain, WOMAC stiffness, 6-min walk	18 months
Stan et al. 2015 [85]	n = 90 Age mean: (i) 67 (c1) 64 (c2) 65 [60+] Female: 70% (in total) Knee osteoarthritis [varus deformity, Ahlback score 3, 4 or 5]	RO	Controlled trial Cost-effectiveness	Payer perspective	QALY (EQ-5D)	Uncertain
Tan et al. 2010 [89]	n = 131 Mean age: (i) 25 (c) 23 Female: (i) 65% (c) 64% Patellofemoral pain syndrome	NL	RCT Cost-utility	Healthcare perspective, societal perspective	QALY (EQ-5D)	12 months
van de Graaf et al. 2020 [90]	n = 319 Meniscal tear [non-obstructive]	NL	RCT Cost-effectiveness	Societal perspective	International Knee Documentation Committee, QALY (EQ-5D)	24 months
van der Graaff et al. 2023 [92]	n = 99 Mean age: (i) 36 (c) 34 [18–45] Female: (i) 26% (c) 23% meniscal tear (traumatic)	NL	RCT Cost-utility	Healthcare perspective, societal perspective	QALY (EQ-5D)	24 months
Others/mixed						
A Abbott et al. 2019 [13] B Pinto et al. 2013 [74]	n = 206 Mean age: (i1) 67 (i2) 67 (i3) 66 (c) 66 Female: (i1) 32% (i2) 28% (i3) 29% (c) 25% Hip osteoarthritis, knee osteoarthritis	NZ	A RCT (MOA-RCT) Cost-effectiveness B RCT (MOA-RCT) Cost-effectiveness, cost-utility	A Societal perspective B Healthcare perspective, societal perspective	A QALY (SF-6D) B OMERACT-OARSI responder, QALY (SF-12v2), WOMAC	A 24 months B 12 months
Bulthuis et al. 2008 [25]	n = 85 Mean age: (i) 69 (c) 69 Female: (i) 42% (c) 28% Hip osteoarthritis, knee osteoarthritis	NL	RCT (DAPPER-study) Cost-utility, cost-effectiveness	Societal perspective	Functional ability, MACTAR and EPMROM	6 months
Coupé et al.‚ 2007 [31]	n = 200 Female: (i) 75% (c) 79% Hip osteoarthritis, knee osteoarthritis	NL	RCT Cost-effectiveness	Societal perspective	QALY (EQ-5D)	15 months
Fernandes et al. 2017 [97]	n = 165 Mean age: (i) 68 (c) 67 Total hip replacement, total knee replacement	DK	RCT Cost-utility	Healthcare perspective	HOOS, KOOS, QALY (EQ-5D)	12 months
Lin et al. 2008 [66]	n = 94 Mean age: (i) 43 (c) 41 Female: (i) 26% (c) 17% Ankle fracture [treated with cast immobilization, with or without surgery before]	AU	RCT Cost-effectiveness	Healthcare perspective, patient perspective	Assessment of Quality of Life, Lower Extremity Functional Scale	5,5 months
*Other conditions*						
Barnhoorn et al. 2018 [19]	n = 56 Mean age: 44 [18–80] Complex regional pain syndrome type 1	NL	RCT Cost-effectiveness	Healthcare perspective, travel costs	QALY (EQ-5D)	9 months
Daker-White et al. 1999 [33]	n = 481 Musculoskeletal problems	UK	RCT Cost-effectiveness	Healthcare perspective, patient perspective	Disease Repercussions Profile, Hospital Anxiety and Depression Scale, Pain—Visual Analogue Scale, SF-36	5–6 months
Heij et al. 2022 [44]	n = 292 Female: (i) 60 (c) 62 Mean age: (i) 82 (c) 81 Mobility problems	NL	RCT Cost-consequences, cost-utility	Healthcare perspective	QALY (EQ-5D)	6 months
Lilje et al. 2014 [65]	n = 78 Mean age: (i) 38 (c) 45 Mixed (on a waiting list for surgery regarding neck, shoulder/arm, back, pelvis/hip, knee or leg/foot condition)+	SE	RCT Cost-consequences	Healthcare perspective	QALY (SF-6D)	12 months
Sevick et al. 2000 (life) [79]	n = 235(+) Sedentary adults	US	RCT (Project ACTIVE) Cost-effectiveness	Practicing clinician	Blood pressure, heart rate, peak VO 2 (mL/kg/min), Physical Activity Recall), total treadmill time, weight	6 months, 24 months
Van den Hout et al. 2005 [91]	n = 300 Female: 79% Rheumatoid arthritis	NL	RCT (RAPIT-study) Cost-utility	Societal perspective	HAQ, MACTAR, QALY (EQ-5D, SF-6D, VAS)	24 months

+information found in an additional paper, ~assumption of the authors, […] inclusion criteria, —not applicable, NA not available, (i) intervention, (c) control intervention, A, B publications based on the same conducted study

*AU* Australia, *DE* Germany, *DK* Denmark, *ES* Spain, *FI* Finland, *GH* Ghana, *IE* Ireland, *IL* Israel, *KR* South-Korea, *NL* Netherlands, *NZ* New-Zealand, *RO* Romania, *SE* Sweden, *TW* Taiwan, *UK* United Kingdom, *US* United States of America, *LBP* low back pain, *EPMROM* Escola Paulista de Medicina Range of Motion scale, *EQ* EuroQol, *HAQ* Health assessment Questionnaire, *HOOS* Hip Disability and Osteoarthritis Outcome Score, *KOOS* The Knee Injury and Osteoarthritis Outcome Score, *MACTAR* McMaster Toronto Arthritis Patient Preference Questionnaire, *NPRS* Numeric Pain Rating Scale, *ODI* Oswestry Disability Index, *OMERACT-OARSI* Outcome Measures in Rheumatology-Osteoarthritis Research Society International, *OSW* Osteoporosis Screening in Older Women, PFFQ Pain Free Function Questionnaire, *QALY* Quality-Adjusted Life Years, *RDS* Roland Disability score, *RDQ* Roland‐Morris Disability Questionnaire, *ROM* Range of motion, *SF* Short Form questionnaire, *VAS* Visual Analogue Scale, *WOMAC* The Western Ontario and McMaster Universities Arthritis Index

The outcomes of the included studies needed to involve a clinical outcome as well as the economic outcome costs. The latter was evaluated from a health-payer perspective in 50 cases and in 36 cases from a societal perspective (Table 1). As the numbers indicate, some studies presented costs for both perspectives. The most frequently utilized clinical outcomes were quality-adjusted life-years (QALYs), which were assessed via the EQ-5D (n = 39) and the SF6 or SF12 (n = 9). Disease-specific disability scores were assessed second most often (n = 9), e.g. via the Roland-Morris Disability Questionnaire. Finally, pain intensity was used in five publications. The remaining publications used individual outcome measures such as bothersomeness of symptoms, a stair climbing task, and blood pressure (Table 1).

**Fig. 4:**
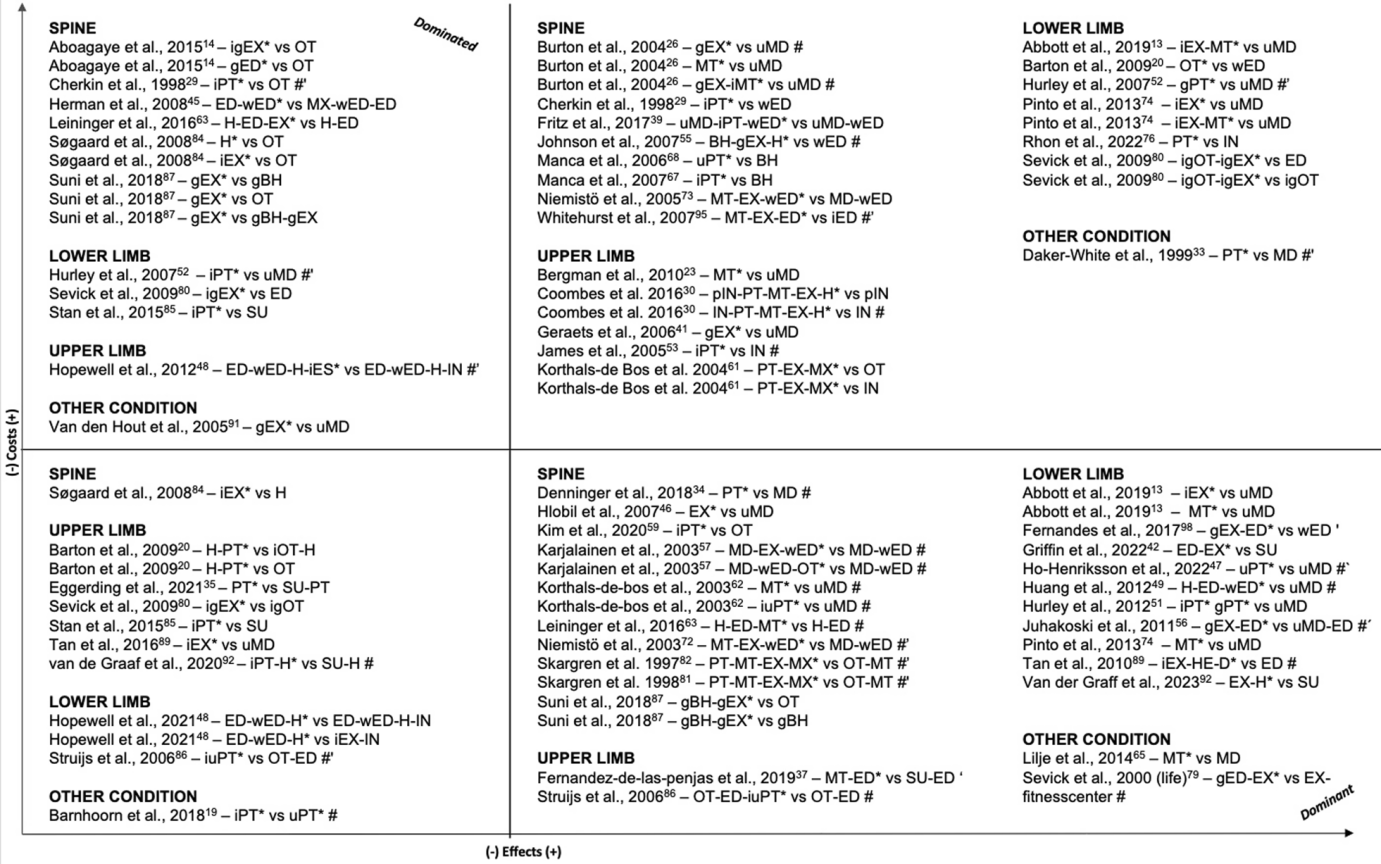
Cost-effectiveness plane of any physiotherapeutic intervention versus another non-physiotherapeutic intervention. BH, behavior; ED, education; EX, exercises; H, home; IN, injection; MD, medical doctor; MT, manual therapy; MX, mixed; OT, others; PT, physiotherapy; SU, surgery; b, booster session; eb, evidence-based; g, group; i, individual; p, placebo; s, stratified care; u, usual; w, written; 2×, twice, *physiotherapeutic intervention, #no significant differences in the health outcome, ‘no significant differences in the costs

**Fig. 5:**
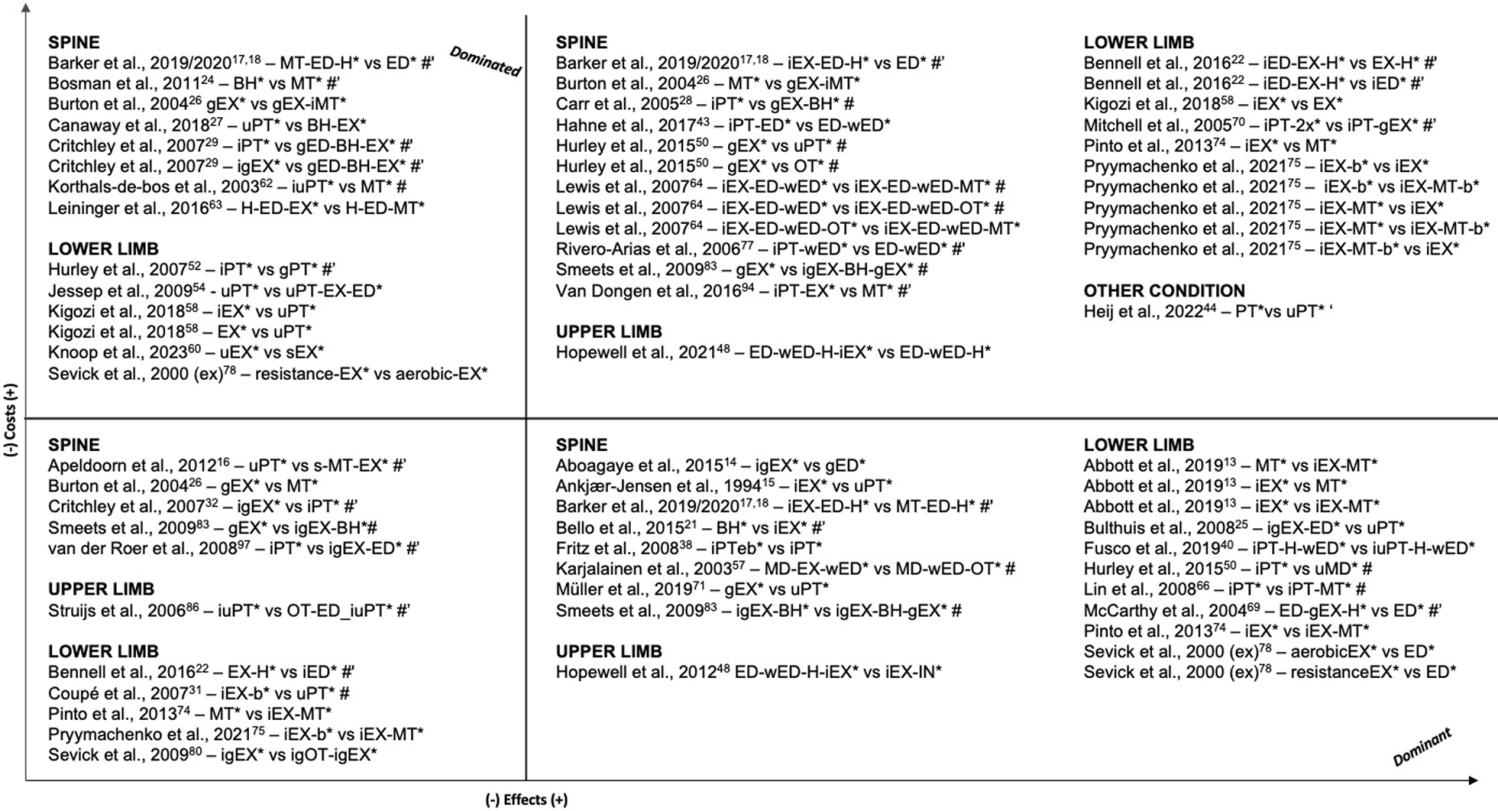
Cost-effectiveness plane of a primary physiotherapeutic intervention vs another physiotherapeutic intervention. BH, behavior; ED, education; EX, exercises; H, home; IN, injection; MD, medical doctor; MT, manual therapy; MX, mixed; OT, others; PT, physiotherapy; SU, surgery; b, booster session; eb, evidence-based; g, group; i, individual; p, placebo; s, stratified care; u, usual; w, written; 2×, twice; *physiotherapeutic intervention, #no significant differences in the health outcome, ‘no significant differences in the costs

In Table 2, we present details on the interventions and highlight our defined primary intervention. The articles involved 150 comparisons between physiotherapeutic interventions and comparators. Figures 4 and 5 display the results in terms of differences in costs and effects grouped qualitatively according to the four quadrants of the cost-effectiveness plane. Eighty-one comparisons involved one treatment provided by a physiotherapist versus another non-physiotherapeutic intervention (Fig. 4), while 69 of the comparisons were between two physiotherapeutic treatments (Fig. 5).

**Table 2:** Overview of interventions provided in the included trial-based economic evaluations

Study ID	Condition, setting	Intervention(s) and comparator(s)			
		Frequency	Intensity	Time	Type
*Spine*					
*Back*					
**A** Barker et al. 2019 [17] **B** Barker et al. 2020 [18]	Osteoporotic vertebral fracture Outpatient, rehabilitation	(i1) Up to 7 sessions over 12 weeks (i2) up to 7 sessions over 12 weeks, home exercises daily (c) 1 session	(i1) yes (i2) yes (c) –	(i1) 1 h assessment, following sessions 30 min (manual therapy), 15 min (stretches) (i2) 1 h assessment, following sessions 30 min (exercise therapy), 45 min/day on 3 to 5 days (home exercises) (c) 1 h	(i1)* manual therapy, home stretching and education (i2)* individual exercise therapy, home exercises and education (c)* education by PT
Müller et al. 2019 [71]	Back pain Outpatient, therapy	(i) 36 sessions over 6 months (week 1–12: 2x/week, week 13–24: 1x/week) (c) NA	(i) yes (c) NA	(i) 1 h (c) NA	(i)* group exercise therapy (c)* usual physiotherapeutic care
Søgaard et al. 2008 [84]	Lumbar spinal fusion Outpatient, rehabilitation	(i1) 3 sessions over 8 weeks (i2) 2x/week over 8 weeks (c) 1 session	(i1) NA (i2) NA (c) –	(i1) 1,5 h (i2) NA (c) NA	(i1) group meetings for interpatient exchange of experiences for the promotion of cooping (i2)* individual exercise therapy (c)* oral instruction for home exercises
*Low back pain*					
Aboagye et al. 2015 [14]	LBP Outpatient, therapy	(i1) 2x/week over 6 weeks, afterwards alone at least 2x/week (i2) over 6 weeks, afterwards biweekly group, and alone at least 2x/week (c) NA	(i1) NA (i2) NA (c) –	(i1) NA (i2) NA (c) NA	(i1) group yoga (i2)* individual + group exercise therapy (c)* evidence-based self-care advice group by back specialist #
Ankjær-Jensen et al. 1994 [15]	LBP (herniated disc) Outpatient, rehabilitation	(i) NA (c) NA	(i) partly (c) NA	(i) NA (c) NA	(i)* group exercise therapy~ (c)* usual physiotherapeutic care~
Apeldoorn et al. 2012 [16]	LBP (chronic) Outpatient, therapy	(i) over 4 weeks minimum, afterwards treatment could change (c) NA	(i) NA (c) NA	(i) NA (c) NA	(i)* stratified treatment (adjusted Delitto´s classified treatment approach): either direction specific exercises, spinal manipulation or stabilization exercises (c)* usual physiotherapeutic care
Bello et al. 2015 [21]	LBP (chronic) Outpatient, therapy	(i) 2x/week over 12 weeks (c) 2x/week over 12 weeks	(i) yes (c) yes	(i) 45 min (c) 45 min	(i)* behavioral graded activity (c)* individual conventional exercise therapy program
Burton et al. 2004 [26]	LBP (non-specific) outpatient, therapy	(i1) up to 9 sessions over 12 weeks (i2) 8 sessions over 12 weeks (i3) i1 and i2 over six weeks (c) –	(i1) NA (i2) NA (c) –	(i1) NA (i2) NA (i3) NA (c) NA	(i1)* group exercise therapy (i2)* spinal manipulation (i3)* group exercise therapy and individual spinal manipulation (c) usual care in GP
Canaway et al. 2018 [27]	LBP Outpatient, therapy	(i) min. 2 sessions in total+ (c) NA	(i) yes (c) NA	(i) initial session of 40 min, following sessions 20–30 min+ (c) NA	(i)* individual behavior changes and exercise therapy (c)* usual physiotherapeutic care
Carr et al. 2005 [28]	LBP Outpatient, therapy	(i) 8 sessions over 4 weeks (c) at the discretion of the physiotherapist	(i) partly (c) NA	(i) 1 h (c) NA	(i)* group exercise therapy incl. cognitive behavioral approach (c)* individual physiotherapy
Cherkin et al. 1998 [29]	LBP Outpatient, therapy	(i1) up to 8 additional sessions (at the discretion of therapist) (i2) up to 8 additional sessions (at the discretion of therapist) (c) –	(i) NA (c) –	(i1) NA (i2) NA (c) –	(i1) chiropractic (i2)* individual physical therapy (McKenzie) (c) education by booklet
Critchley et al. 2007 [32]	LBP (acute) Outpatient, therapy	(i1) up to 8 sessions (i2) up to 8 sessions (c) up to 12 sessions	(i1) partly (i2) partly (c) partly	(i1) 1,5 h (i2) 1,5 h (c) 30 min	(i1)* individual and group spinal stabilization (i2)* group education: cognitive-behavioral approach and light exercises (c)* individual physiotherapy
Fritz et al. 2008 [38]	LBP (acute) Outpatient, therapy	(i) mean: 4.6 sessions over 25.4 days (c) mean: 5.9 sessions over 29.7 days	(i) NA (c) NA	(i) NA (c) NA	(i)* individual physiotherapy following evidence-based guidelines (c)* physiotherapy not following evidence-based guidelines
Fritz et al. 2017 [39]	LBP Outpatient, therapy	(i) 4 sessions over 4 weeks (c) –	(i) NA (c) –	(i) NA (c) NA	(i)* usual primary care, booklet and early individual physiotherapy (c) usual primary care, booklet and waiting min. 4 weeks before considering additional treatments #
Hahne et al. 2017 [43]	LBP (chronic) Inpatient, therapy	(i) 10 sessions over 10 weeks (c) 2 sessions over 10 weeks	(i) partly (c) –	(i) 30 min (c) 30 min	(i)* individual physiotherapy (pathoanatomical, psychosocial, neurophysiological) and education by PT (c)* guideline-based education by PT and booklet
Herman et al. 2008 [45]	LBP Outpatient, therapy	(i) 2x/week over 3 months (c) bi-weekly over 3 months	(i) NA (c) –	(i) 30 min (c) 30 min	(i) individual neuropathic care (acupuncture, exercise and dietary advice, relaxation), education by PT and booklet (c)* standardized education by PT and booklet
Hlobil et al. 2007 [46]	LBP (chronic) Outpatient, therapy	(i) 2x/week over 3 months or until patient can fully return to previous duties (c) –	(i) partly (c) –	(i) 1 h (c) –	(i)* Graded Activity intervention (c) usual care in GP
Hurley et al. 2015 [50]	LBP Outpatient, therapy	(i1) weekly phone contact over 8 weeks (i2) 1 session 1x/week over 8 weeks c) NA	(i1) yes (i2) partly (c) NA	(i1) NA (i2) 1 h (c) NA	(i1)* walking program (i2)* group exercise therapy (c)* usual physiotherapeutic care
Johnson et al. 2007 [55]	LBP Outpatient, therapy	(i) 8 sessions over 6 weeks (c) –	(i) partly (c) –	(i) 2 h (group sessions) (c) –	(i)* cognitive behavorial therapy, group exercise therapy and home exercises (c) education by booklet
Karjalainen et al. 2003 [57]	LBP outpatient/inpatient, therapy	(i1) 1 session (i2) 1 session (c) –	(i1) partly (i2) NA (c) NA	(i1) 1,5 h (i2) 75 min (c) –	(i1)* GP visit, light mobilization, graded activity exercises, leaflet (i2) GP visit, leaflet, visit of the patient’s work site by PT to review how the patient deals with the information given (c) GP visit, leaflet
Kim et al. 2020 [59]	LBP (chronic) outpatient, therapy	(i) 6 sessions over 3 weeks (c) 6 sessions over 3 weeks	(i) NA (c) NA	(i) 20 min (c) 20 min	(i)* individual physical therapy: ultrasound, electrotherapy, hot pack (c) massage chair #
**A** Niemistö et al. 2003 [72] **B** Niemistö et al. 2005 [73]	LBP (chronic) Outpatient, therapy	(i) 4 sessions over 4 weeks (c) reinforced at 5 month follow up	**A** (i) yes (c) – **B** (i) partly (c) –	(i) 1 h (c) –	(i)* individual evaluation, manipulative treatment, exercises and booklet (c) physician consultation, education by booklet about low back pain, including exercise and coping advice
Rivero-Arias et al. 2006 [77]	LBP (chronic) Outpatient, therapy	(i) up to 5 sessions (c) 1 session	(i) NA (c) –	(i) initial session 1 h, following sessions 30 min (c) 1 h	(i)* individual physiotherapy and education by booklet (c)* education by PT and booklet
Smeets et al. 2009 [83]	LBP Outpatient, therapy	all: 10 weeks intervention (i1) *GA:* 1-3x/week, 20 sessions (3 group, 17 individual) *PST:* 10 sessions (i2) PST: 10 sessions *GA:* start at 3 weeks, 19 sessions APT: 3x/week, 30 min bicycle and 75 min strength/endurance training (c) APT: 3x/week, 30 min bicycle and 75 min strength/endurance training	(i1) partly (i2) i1 and c combined (c) yes	(i1) GA: 30 min PST: 1,5 h (i2) APT: 1,75 h PST: 1,5 h GA: 30 min (c) 1,75 h	(i1)* group and individual graded activity (GA) with problem solving (PST) (i2)* c and i1 combined (c)* group active physical treatment (APT) #
Suni et al. 2018 [87]	LBP (chronic) Outpatient, therapy	(i1) i2 and i3 combined (i2) in total: 32 sessions; 2x/week over 8 weeks, followed by 1x/week instructed session and 1x/week home session over 16 weeks (i3) 4 sessions 1x/week over 4 weeks, followed by 6 sessions every third week for 24 weeks	(i1) yes (i2) yes (i3) yes (c) – +	(i1) 1,75 h (i2) 1 h (i3) 45 min	(i1)* i2 and i3 combined (i2)* group exercise therapy (i3) group counseling based on cognitive behavioral learning (c) wait and see
van der Roer et al. 2008 [93]	LBP Outpatient, therapy	(i) in total: 46 sessions over 30 weeks including 3 phases, 1. phase—10 individual sessions and 20 group sessions over 3 weeks 2. phase—group sessions 2x/week over 8 weeks 3. phase—decreased frequency of sessions, more home exercises (c) number of treatment sessions at discretion of the PTs, on average 9 sessions over 6 weeks +	(i) yes (c) yes	(i) individual sessions: 30 min, group sessions: 1,5 h (c) NA	(i)* group and individual exercise therapy and education by back school according to behavioral principles (c)* individual physiotherapy according to the Low Back Pain Guidelines of the Royal Dutch College for Physiotherapy #
Whitehurst et al. 2007 [95]	LBP Outpatient, therapy	(i) 1 initial session + up to 6 sessions following (c) 1 initial session + up to 6 sessions following	(i) partly (c) –	(i) initial session 40 min, following sessions 20 min (c) initial session 40 min, following sessions 20 min	(i)* manual therapy, individual back-specific exercises, advice (c)* individual brief pain management based on the biopsychosocial model of care #
*Neck*					
Bosmans et al. 2011 [24]	Neck pain (subacute) Outpatient, therapy	(i) up to 18 sessions (c) up to 6 sessions over 6 weeks	(i) partly (c) NA	(i) 30 min (c) 30–45 min	(i)* Behavioral Graded Activity (c)* manual therapy
Korthals-de Bos et al. 2003 [62]	Neck pain Outpatient, therapy	(i1) up to 6 sessions 1x/week over 6 weeks (i2) up to 12 sessions 2x/week (c) –	(i1) partly (i2) NA (c) –	(i1) 45 min (i2) 30 min (c) –	(i1)* manual therapy (i2)* individual physiotherapy (exercises, optional massage or partly manual therapy) (c) usual care in GP
Leininger et al. 2016 [63]	Neck pain (chronic) Outpatient, therapy	(i1) 4 sessions of education and up to 20 sessions of SMT over 12 weeks (i2) 4 sessions of education and 20 sessions of SRE over 12 weeks (c) 4 sessions over 12 weeks	(i) yes (c) NA	(i1) 1 h (i2) 1 h (c) 1 h	(i1)* home exercises and advice (HEA) and spinal manipulative therapy (SMT) (i2)* HEA and individual supervised rehabilitative exercises (SRE) (c) home exercise and advice (HEA) #
Lewis et al. 2007 [64]	Neck disorders (non-specific) Outpatient, therapy	(i1) mean: 5.79 sessions followed by up to 6 sessions over 6 weeks (i2) mean: 6.63 sessions followed by up to 6 sessions over 6 weeks (c) mean: 4.49 sessions followed by up to 6 sessions over 6 weeks	(i1) partly (i2) partly (c) partly +	(i1) initial session 40 min, following sessions 20 min (i2) initial session 40 min, following sessions 20 min (c) initial session 40 min, following sessions 20 min	(i1)* c and manual therapy (i2)* c and pulsed shortwave diathermy c)* individual exercise therapy, advice by PT and booklet (Arthritis Research Campaign's “Pain in the Neck” booklet)
Manca et al. 2006 [68]	Neck pain Outpatient, therapy	(i) 1–3 sessions (c) according to individual judgment of PT	(i) – (c) NA	(i) NA (c) NA	(i) cognitive-behavioral treatment c)* usual physiotherapeutic care (electrotherapy, manual therapy, advice, acupuncture, other treatments)
Van Dongen et al. 2016 [94]	Neck pain (subacute and chronic) Outpatient, therapy	(i) up to 6 sessions 1x/week or bi-weekly, determined by PT (c) up to 9 sessions up to 2x/week	(i) NA (c) NA	(i) 30 min—1 h (c) 30 min	(i)* Manual Therapy according to the Utrecht school c)* individual physiotherapy, with at least 20 min of active exercises #
*Others/mixed*					
Denninger et al. 2018 [34]	Back or neck pain Outpatient, therapy	(i) mean: 7 sessions (c) mean: 8 sessions	(i) NA (c) NA	(i) NA (c) NA	(i)* individual initial contact by direct access to a PT (back and neck program) and following physical therapy (c) initial contact by traditional medical referral #
Manca et al. 2007 [67]	Back or neck pain Outpatient, therapy	(i) mean: 3,1 sessions (SD:2,5), range: 0 to 7+ (c) mean: 4,15 sessions (SD:2,8), range: 0 to 7+	(i) NA (c) NA	(i) NA (c) NA	(i) individual Solution Finding Approach c)* individual McKenzie therapy #
**A** Skargren et al. 1997 [82] **B** Skargren et al. 1998 [81]	Back or neck pain Outpatient, therapy	(i) mean: 6 sessions (c) mean: 5 sessions	(i) NA (c) NA	(i) NA (c) NA	(i)* physiotherapy (manipulation, mobilization, traction, soft tissue treatment, McKenzie treatment, TENS, acupuncture, relaxation training, training program) (c) chiropractic (manipulation, mobilization, traction, soft tissue treatment)
*Upper Limb*					
Bergman et al. 2010 [23]	Shoulder complaints Outpatient, therapy	(i) up to 6 sessions over 12 weeks (c) –	(i) yes (c) –	(i) NA (c) –	(i)* manual therapy (manipulative and mobilization of the cervicothoracic spine and adjacent ribs) (c) usual care in GP
Commbes et al. 2016 [30]	Epicondylitis lateralis [> 6 weeks duration] Outpatient, therapy	(i1) 8 sessions over 8 weeks (physiotherapy) (i2) 8 sessions over 8 weeks (physiotherapy) (c1) 1 session (c2) 1 session	(i1) yes (i2) yes (c1) yes (c2) yes	(i1) 30 min (i2) 30 min (c1) – (c2) –	(i1)* saline injection (placebo) followed by physiotherapy (manual therapy, exercise, home exercises) (i2) corticosteroid injection followed by physiotherapy (manual therapy, exercise, home exercises) c1) saline injection (placebo) # c2) corticosteroid injection #
Fernandez-de-Las-penjas et al. 2019 [37]	Carpal tunnel syndrome Outpatient, therapy	(i) 3 sessions 1x/week (c) –	(i) yes (c) –	(i) 30 min (c) –	(i)* manual therapy and education for exercises (c) open or endoscopic surgery and education for exercises
Geraets et al. 2006 [41]	Shoulder complaints (chronic) Outpatient, therapy	(i) up to 18 sessions over 12 weeks (c) –	(i) yes (c) –	(i) 1 h (c) –	(i)* group graded exercise therapy (c) usual care in GP
Hopewell et al. 2021 [48]	Rotator cuff disorder Outpatient, therapy	(i1) injection and c (i2) up to 6 sessions over 16 weeks (i3) injection and i2 (c) 1 session	(i1) – (i2) partly (i2) partly (c) –	(i1) 1 h (i2) initial session 1 h, following sessions 20–30 min (i3) injection and i2 (c) 1 h	(i1) c and corticosteroid injection (i2)* c and individual exercise therapy (i3) individual exercise therapy and corticosteroid injection (c)* best-practice advice by PT, education by booklet and home exercises #
James et al. 2005 [53]	Shoulder pain Outpatient, therapy	(i) up to 8 sessions over 6 weeks (c) 1 injection at beginning, if symptoms persisted patients could have 1 additional injection within 4 weeks	(i) NA (c) –	(i) 20 min (c) –	(i)* individual ( ~) physiotherapy (c) corticosteroid injection into subacromial space #
Korthals-de Bos et al. 2004 [61]	Epicondylitis lateralis Outpatient, therapy	(i) max. 9 sessions 2x/week over 6 weeks (c1) 1 session (c2) 1 session	(i) partly (c1) yes (c) –	(i) 30 min (c1) NA (c2) NA	(i)* physiotherapy (ultrasound, deep friction massage, exercise) (c1) corticosteroid injections # (c2) wait-and-see #
Struijs et al. 2006 [86]	Epicondylitis lateralis Outpatient, therapy	(i1) 9 sessions over 6 weeks (i2) i1 and c (c) over 6 weeks	(i1) yes (i2) yes (c) –	(i1) 30 min (7,5 min ultrasound, 5–10 min friction) (i2) i1 and c combined (c) wearing the brace continuously during the day	(i1)* individual physiotherapy (ultrasound, friction massage, home exercises, if pain subsided) (i2)* i1 and c (c) brace, 1 initial PT visit for instruction
*Lower limb*					
*Hip*					
Fusco et al. 2019 [40]	Hip replacement Inpatient, rehabilitation	(i), (c) 2x/day until hospital discharge, 8 weeks home exercises, 2 weeks after discharge 1 session at home or outpatient	(i) NA (c) NA	(i) NA (c) NA	(i)* individual physiotherapy without 'hip precautions' and home exercises via booklet (c)* individual physiotherapy with 'hip precautions' and home exercises via booklet
Griffin et al. 2022 [42]	Femoroacetabular Impingement syndrome Outpatient, therapy	(i) – (c) 6–10 sessions over 12–24 weeks	(i) – (c) yes	(i) – (c) mean: 30 min	(i) hip arthroscopy c)* best conservative care (personalized hip therapy: education and exercise therapy, sometimes per telephone or e-mail)
Juhakoski et al. 2011 [56]	Hip osteoarthritis Outpatient, therapy	(i) 1 session (education), 12 sessions 1x/week, after that 3x/week home exercise over 2 years, 4 booster sessions 1 year later (exercise) (c) 1 session (education)	(i) yes (c) –	(i) 1 h (education), 45 min (exercise) (c) 1 h (education)	(i)* group exercise therapy and education by physician (c) usual care in GP and education by physician
Tan et al. 2016 [88]	Hip osteoarthritis Outpatient, therapy	(i) max. 12 sessions over the first 3 months, followed by 3 booster sessions at month 5, 6 and 9 (c) –	(i) NA (c) –	(i) NA (c) –	(i)* individual ( ~) exercise therapy (c) usual care in GP
*Knee*					
Barton et al. 2009 [20]	Knee pain Outpatient, therapy	(i1) *dietary intervention*: 15 sessions over 24 months (monthly until 6th month, then every other month) *home exercises*: daily, 6 sessions with a PT every 4 months over 24 months (i2) 15 sessions over 24 (i3) daily, 6 sessions with a PT over 24 months (c) –	(i1) *home exercises:* partly (i2) – (i3) partly (c) –	(i1) NA (i2) NA (i3) NA (c) –	(i1) individual dietary intervention and quadriceps strengthening home exercises (i2) individual dietary intervention (i3)* quadriceps strengthening home exercises with PT visits (c) education by leaflet
Bennell et al. 2016 [22]	Knee osteoarthritis Outpatient, therapy	(i) 10 sessions over 12 weeks, home program: 4x/week over 12 weeks, followed by 3x/week over 9 months (c1) exercise therapy: 10 sessions home program: 4x/week over 12 weeks, followed by 3x/week over 9 months (c2) 10 sessions 1x/week	(i) partly (c1) partly (c2) –	(i) 70 min (c1) 25 min (exercise therapy) (c2) 45 min	(i)* individual education (pain coping) and exercise therapy and home program (c1)* exercise therapy and home program only (c2)* individual education (pain coping) by PT #
Eggerding et al. 2021 [35]	ACL tear Outpatient, rehabilitation	(i) until good functional control was achieved + (c) according to the recommendations of the Dutch ACL guideline, min. 3 months	(i) NA (c) NA	(i) NA (c) NA	(i) early ACL reconstruction, within six weeks after randomization, after that referred for individual physical therapy + (c)* supervised individual physical therapy program, then optional reconstruction+#
Ho-Henriksson et al. 2022 [47]	Knee osteoarthritis Outpatient, therapy	(i) mean: 4 individual and two group sessions; 0,3 physician visits (c) mean: 4 individual and 1,5 group sessions; 1,5 physician visits	(i) NA (c) NA	(i) NA (c) NA	(i)* primary access to individual PT (education, exercise therapy, pain treatment, walking aids) and group treatment (BOA-program: education, exercise therapy) (c) primary access to physician (education, medical prescription, referrals)
Huang et al. 2012 [49]	Total knee replacement Outpatient, prehabilitation	(i) daily, over 4 weeks before surgery (c) NA	(i) NA (c) NA	(i) 40 min/day (c) NA	(i)* home exercises and education before replacement by PT and booklet (c) conventional pre-TKA care
**A** Hurley et al. 2007 [52] **B** Hurley et al. 2012 [51]	Knee pain (chronic) Outpatient, therapy	(i1) 12 sessions 2x/week over 6 weeks (i2) 12 sessions 2x/week over 6 weeks (c) –	(i1) partly (i2) partly (c) –	(i1) 15–20 min (i2) 15–20 min (c) –	(i1)* ESCAPE program (exercise and self-management education) (i2)* group rehabilitation program (c) usual primary care
Jessep et al. 2009 [54]	Knee pain (chronic) Outpatient, therapy	(i) 10 sessions 2x/week over 5 weeks plus booster at 4 month plus home exercises (c) mean: 4 sessions	(i) partly (c) NA	(i) approx. 60 min (c) NA	(i)* adapted ESCAPE program (exercise and self-management education) (c)* usual care by PT (exercise, advice, electrotherapy, MT)
Kigozi et al. 2018 [58]	Knee osteoarthritis Outpatient, therapy	(i1) 6–8 sessions over 12 weeks (i2) 4 sessions until week 12, 4–6 follow-ups until sixth month (c) up to 4 sessions over 12 weeks	(i1) partly (i2) partly (c) NA	(i1) NA (i2) NA (c) NA	(i1)* individual exercise program (i2)* targeted exercise adherence (c)* usual physiotherapeutic care (individual exercise therapy and education by booklet)
Knoop et al. 2023 [60]	Knee osteoarthritis Outpatient, therapy	(i) 3–18 sessions individual over 12 weeks plus 1–3 booster sessions (c) mean: 10 sessions over 12 weeks	(i) NA (c) NA	(i) NA (c) NA	(i)* stratified exercise therapy (c)* usual exercise therapy by PT
McCarthy et al. 2004 [69]	Knee osteoarthritis Outpatient, therapy	(i) 1 initial session (education), 2x/week over 8 weeks (exercise) (c) 1 initial session (education)	(i) yes (exercise) (c) –	(i) 45 min (exercise) (c) NA	(i)* c, group exercise therapy and home exercises (c)* education by PT based on the Research Campaign's information booklet 'Osteoarthritis of the knee' and home exercises #
Mitchell et al. 2005 [70]	Total knee replacement Outpatient/home, prehabilitation/rehabilitation	(i) min. 3 pre-TKR sessions and up to 6 post-discharge sessions (c) 1-2x/week (group exercise) and at PT's discretion (individual)	(i) NA (c) NA	(i) NA (c) NA	(i)* individual physiotherapy at home pre- and post-TKA (c)* individual physiotherapy and group exercise therapy post-TKA only
Pryymachenko et al. 2021 [75]	Knee osteoarthritis Outpatient, therapy	(i1) 12 sessions over 1 year (i2) 12 sessions over 9 weeks (i3) 12 sessions over 1 year (c) over 9 weeks	(i1) partly (i2) partly (i3) partly (c) partly	(i1) NA (i2) NA (i3) NA (c) NA	(i1)* individual exercise therapy and booster session (i2)* individual exercise therapy and manual therapy (i3)* individual exercise therapy, manual therapy and booster session (c)* individual exercise therapy #
Rhon et al. 2022 [76]	Knee osteoarthritis Outpatient, therapy +	(i) 8 sessions over 4–6 weeks, additional 3 sessions between 4 and 9th month (c) 1 session	(i) yes (c) –	(i) 1 h (c) –	(i)* physical therapy (exercises, joint mobilization) (c) glucocorticoid injection
Sevick et al. 2000 (ex) [78]	Knee osteoarthritis Outpatient, therapy	(i1), (i2) month 1–3: 3x/week of 1 h month 4–6: home exercises of 1 h, bi-weekly contact to PT (4 home visits, 6 telephone calls) month 7–9: home exercises of 1 h, every 3 weeks contact to PT (telephone calls) month 10–18: 1x/month contact to PT (telephone calls) (c) month 1–3: 3 sessions of 1,5 h month 4–6: biweekly nurse contact month 7–18: 1x/month nurse contact	(i1) yes (i2) yes (c) –	(i1) and (i2) month 1–3: 1 h month 4–6: home exercises of 1 h month 7–9: home exercises of 1 h month 10–18: NA month 1–3: 1,5 h month 4–6: NA month 7–18: NA	(i1)* aerobic exercise training (3 months in a group, 15 months homebased individual) (i2)* resistance exercise (3 months in a group, 15 months homebased individual) (c)* health education
Sevick et al. 2009 [80]	Knee osteoarthritis Outpatient, therapy	(i1) 1 initial visit at home, months 1–4: monthly 3 group sessions, 1 individual session months 5–6: biweekly 3 group sessions, 1 individual months 7+: biweekly telephone call or meeting and newsletter (i2) 3x/week over 4 months, after 4 month decision: continue at facility, at home or combined (i3) i1 and i2 combined (c) months 1–3: monthly meeting months 4–5: monthly phone contact months 5+: bimonthly contact	(i1) – (i2) yes (i3) i1 and i2 combined (c) –	(i1) 1 initial visit at home, months 1–4: NA months 5–6: NA months 7+: NA (i2) 60 min over 4 months (i3) i1 and i2 combined (c) months 1–3: 1 h months 4–5: NA months 5+: NA	(i1) diet: group and individual (i2)* group and individual exercise therapy (i3)* i1 and i2 combined (c) healthy lifestyle control
Stan et al. 2015 [85]	Knee osteoarthritis Inpatient, therapy/operation	(i) exercises and following PT session, 2x/day over 5 days before discharging c1) – c2) –	(i) NA c1) – c2) –	(i) 30 min (exercises) c1) – c2) –	(i)* individual rehabilitation program c1) total knee arthroplasty c2) total knee arthroplasty following high tibial osteotomy #
Tan et al. 2010 [89]	Patellofemoral pain syndrome Outpatient, therapy	(i) 9 sessions over 6 weeks (c) NA	(i) NA (c) NA	(i) NA (c) NA	(i)* individual exercise therapy and home exercises and c-intervention (c) education by a physician
Van de Graaf et al. 2020 [90]	Meniscal tear Outpatient, therapy	(i) 16 sessions over 8 weeks, 2x/week home exercises (c) –	(i) yes (c) –	(i) 30 min (individual PT) (c) –	(i)* individual physiotherapy and home exercises (c) arthroscopic partial meniscectomy and same home exercises as i #
Van der Graaff et al. 2023 [92]	Meniscal tear (traumatic) Outpatient, rehabilitation	(i) NA (c) NA	(i) partly	(i) NA (c) –	(i)* exercise programme, home exercises (c) arthroscopic partial meniscectomy
*Others/mixed*					
**A** Abbott et al. 2019 [13] **B** Pinto et al. 2013 [74]	Hip osteoarthritis, knee osteoarthritis Outpatient, therapy	(i1), (i2) and (i3) 7 sessions over 9 weeks, 2 booster sessions at week 16 and 54 (c) –	**A** (i1) NA (i2) NA (i3) NA (c) – **B** (i1) partly (i2) partly (i3) partly (c) –	(i1) approx. 50 min (i2) approx. 50 min (i3) approx. 50 min (c) –	(i1)* individual exercise therapy (i2)* manual therapy (i3)* individual exercise and manual therapy (c) usual care in GP
Bulthuis et al. 2008 [25]	Hip osteoarthritis, knee osteoarthritis Inpatient, rehabilitation	(i) 2x/day over 3 weeks (exercise therapy), 2x/week over 3 weeks (education) (c) NA	(i) partly (c) NA	(i) 75 min (exercise therapy) (c) NA	(i)* individual and group exercise therapy and education by PT (c)* usual PT-care
Coupé et al.‚ 2007 [31]	Hip osteoarthritis, knee osteoarthritis Outpatient, therapy	(i) max. 18 session over 12 weeks and max. 7 booster sessions (c) max. 18 sessions within 12 weeks	(i) partly (c) NA	(i) NA (c) NA	(i)* individual exercise therapy and booster session (c)* usual physiotherapeutic care
Fernandes et al. 2017 [97]	Total hip replacement, total knee replacement Outpatient, prehabilitation	(i) 2x/week over 8 weeks (c) –	(i) partly (c) –	(i) 1 h (c) –	(i)* group neuromuscular exercise program and education by PT before surgery (c) standard preoperative information by leaflet
Lin et al. 2008 [66]	Ankle fracture Outpatient, therapy	(i) mean no. of treatment: 10 sessions, 2x/week over 4 weeks (c) first week: 2 sessions, after that 1x/week, mean number of sessions: 6	(i) yes (c) partly	(i) NA (c) NA	(i)* individual physiotherapy and manual therapy (c)* individual physiotherapy
*Other conditions*					
Barnhoorn et al. 2018 [19]	Complex regional pain syndrome type 1 Outpatient, therapy	(i) max. 5 sessions (c) –	(i) NA (c) –	(i) 40 min (c) –	(i)* individual pain exposure physical therapy (c)* usual physiotherapeutic care+
Daker-White et al. 1999 [33]	Musculoskeletal problems Outpatient, therapy	(i) – (c) –	(i) – (c) –	(i) NA (c) NA	(i)* treatment by PT (orthopedic specialist) (c) treatment by orthopedic surgeons #
Heij et al. 2022 [44]	Mobility problems Outpatient, therapy	(i) mean: 15 sessions (c) mean: 22 sessions	(i) partly+	(i) 30 min+	(i)* physical therapy (Coach2move) (c)* usual physiotherapeutic care
Lilje et al. 2014 [65]	Mixed (on a waiting list for surgery regarding neck, shoulder/arm, back, pelvis/hip, knee or leg/foot condition)+ Outpatient, therapy	(i) up to 5 sessions over 5 weeks (c) as many appointments as required	(i) NA (c) –	(i) 30–45 min (c) NA	(i)* manual therapy (c) standard care of orthopedic surgeons
Sevick et al. 2000 (life) [79]	Sedentary adults Outpatient, therapy	(i) 1x/week (1–16 week), biweekly (17–24 week), 1x/month group meeting (7–12 months), every other month group meeting (13–18 months), quarterly group meeting (19–24 months) (c) exercise (1–6 months), receiving a calendar—monthly, invitation to activities, newsletter: quarterly (7–18 months)	(i) partly (c) yes	(i) NA (c) 20 min—1 h (exercise)	(i)* group education and lifestyle exercise therapy (for 6 months), individual/group—education, life exercise therapy (7–18 months) (c) 1–6 months: individual exercises at a fitness facility 7–18 months: patients had the choice if they want to continue exercising at a fitness facility, calendar of activities, invitation to activities and newsletter #
Van den Hout et al. 2005 [91]	Rheumatoid arthritis Outpatient, therapy	(i) 2x/week over 2 years, in total: 60 sessions (c) mean sessions of individual PT: 8.4 over 2 years	(i) partly (c) –	(i) 75 min (c) NA	(i)* group exercise therapy ('Rheumatoid Arthritis Patients in Training'-program: weight bearing) (c) usual care in GP (individual physiotherapy if necessary)

^**#**^ control group defined by authors, +information found in an additional paper, ~assumption of the authors, […] inclusion criteria, —not applicable, *physiotherapeutic treatment, NA not available, (i) intervention, (c) control intervention, A, B publications based on the same conducted study, bold printed description of one (control-) intervention the authors’ treatment of interest

*ACL* anterior cruciate ligament, *GP* general practice, *LBP* low back pain, *PT* physiotherapist(s), *prehabilitation* physiotherapeutic treatment before a scheduled surgery, *rehabilitation* physiotherapeutic treatment after a surgery or traumatic injury, *therapy* physiotherapeutic treatment of a degenerative disease or an inflammatory disease

### Critical Appraisal of the Included Articles

More than two-thirds of the included 83 trials-based economic evaluations were of high quality with sum-yes-scores of 17 (n = 13), 18 (n = 16), or 19 (n = 31) on the Consensus on Health Economic Criteria checklist (Additional file 3: Table S3). The lowest score was 11 which was observed in two articles. Scores of 12, 13, and 14 were observed in two articles each. The scores 15, and 16 were reached by five and ten articles, respectively. The three items “Is a well-defined research question posed in answerable form?”, “Is the actual perspective chosen appropriate?”, and “Are all outcomes measured appropriately?” were evaluated with a ‘Yes’ for all the articles. The lowest sum of positive evaluation was observed for the item “Does the article indicate that there was no potential conflict of interest of study researchers and funders?” (n = 58). Limitations were also observed in 23 articles when it comes to the performance of incremental analyses of the costs and outcomes of alternatives. Fifty-nine publications met the criteria regarding sensitivity analyses to account for uncertain variable values. Nonetheless, the overall article quality was high.

### Results of Syntheses Grouped by Body Parts

#### Spine

Of our 39 articles dealing with spine-related conditions four address the back in general [17, 18, 71, 84], 25 deal with the low back [14–16, 21, 26–29, 32, 38, 39, 43, 45, 46, 50, 55, 57, 59, 72, 73, 77, 83, 87, 93, 95], six with the neck [24, 62–64, 68, 94] and four include mixed patients [34, 67, 81, 82] (Tables 1, 2).

The four articles on general back conditions [17, 18, 71, 84] built on three clinical trials. The sessions varied between 1 and 36 over a time horizon of 1 to 24 weeks. Most interventions were conducted 1–2 times per week. The duration if indicated varied between 60 and 90 min. The intervention and control groups contained individual and group-based exercise therapy, manual therapy, and education as well as usual care (Table 2). Group exercise therapy was deemed cost-effective over usual care [71] and interpatient exchange group meetings were cost-effective over increasing the frequency of traditional therapy according to the authors [84]. The articles from Barker et al*.* considered three interventions provided by physiotherapists [17, 18]. The findings of all cost-effectiveness comparisons can be found in Figs. 4 and 5.

The 25 articles on low back pain were built on 24 clinical trials [14–16, 21, 26–29, 32, 38, 39, 43, 45, 46, 50, 55, 57, 59, 72, 73, 77, 83, 87, 93, 95]. Niemistö et al*.* published two articles based on the same clinical trial [72, 73]. Patients received between 1 and 46 sessions lasting between 20 and 120 min, spread out over a maximum of 30 weeks. Sessions were most often offered 1 to 3 times a week. The treatments in the intervention and control groups included mostly group and individual exercise therapy, individual physiotherapeutic treatments, and education. Additional manual therapy, McKenzie-based treatments, behavioral graded activity, behavior change techniques, a walking program, visits at the workplace, yoga, usual care, chiropractic treatment, and a massage chair were provided (Table 2). Fritz et al*.* compared individual physical therapy following evidence-based guidelines to physical therapy not following those guidelines and found that following evidence-based guidelines is cost-effective [38]. The articles on low back pain included a total of 67 comparisons of interventions of which 34 were between a physiotherapeutic intervention and another treatment (Figs. 4, 5).

Of the 6 articles on neck conditions all addressed neck pain [24, 62–64, 68, 94]. One to 24 sessions were offered. They lasted between 20 and 60 min and were spread over up to 12 weeks. Most treatments were offered 1–2 times per week. The intervention contained exercises, cognitive behavioral treatment, behavior-graded activity, manual therapy, individual therapy, and advice. The control group received exercises, usual care involving physiotherapeutic treatments, and written information (Table 2). The articles involve 12 treatment comparisons. Five highlighted better outcomes at higher costs for the physiotherapeutic intervention, and three were dominant. In the four dominated investigations three of the comparison interventions were offered by physiotherapists (Figs. 4, 5).

Two of the four articles with a mixed spine population originated from the same study. These studies did not differentiate between neck and back pain [34, 67, 81, 82]. Participants received up to 8 sessions. One article focused on physiotherapeutic care in comparison to traditional medical referral care, another compared the McKenzie treatment concept to the individual solution-finding approach, and the remaining two, from the same study, compared physiotherapeutic to chiropractic care (Table 2). Considering only the effectiveness, all articles favored the intervention offered by physiotherapists, although not significantly in the articles by Skargren et al. [81,82]. In the article of Manca et al*.* the psychotherapeutic treatments involved higher costs [67]; dominance of the physiotherapeutic treatment was found in the remaining articles [34, 81, 82] (Figs. 4, 5).

#### Upper Limb

The seven papers addressing the upper limb dealt with carpal tunnel syndrome [37], epicondylitis lateralis [61, 86], and shoulder complaints [23, 41, 53] involving a rotator cuff disorder [48]. The number of intervention and control group sessions varied between 3 and 18. Patients were treated 1–2 times a week for 20 to 60 min. All but three articles involved one intervention and one control group. The exceptions involved three different intervention groups. The provided interventions contained manual therapy, education, exercises, and physiotherapeutic treatments such as friction, ultrasound, and massage. The control groups received surgery, injections, braces, and usual care. One study also included a wait-and-see approach (Table 2). The articles involve 14 treatment comparisons. The physiotherapeutic treatment for carpal tunnel syndrome was dominant [37]. The remaining comparison of physiotherapeutic interventions, but one, may as well be cost-effective, depending on the willingness to pay (Figs. 4, 5).

#### Lower Limb

The included 30 articles built on 28 clinical trials dealing with the lower limb. Of these trials, four addressed the hip [40, 42, 56, 88], nineteen the knee [20, 22, 35, 47, 49, 51, 52, 54, 58, 60, 69, 70, 75, 78, 80, 85, 88, 92], and five a mixed population [13, 25, 31, 36, 66, 74]. Nineteen of the clinical trials dealt with osteoarthritis including the last therapy option of a joint replacement [13, 22, 25, 31, 36, 40, 47, 49, 56, 58, 60, 69, 70, 75, 76, 78, 80, 85, 88].

Among the hip-related clinical trials, three dealt with osteoarthritis and one addressed the femoroacetabular impingement syndrome [42]. Patients participated in 1 to 12 sessions. The duration of a session ranged from 30 to 60 min. The frequency varied between twice a day in the inpatient setting and one time in 2 weeks. The treatments offered contained individual as well as group exercises and education. The control groups received the usual care or an arthroscopy. Besides that, Fusco et al*.* compared the cost-effectiveness of a treatment with or without limiting hip motion [40] (Table 2). They conclude that not limiting hip motions after a hip replacement is dominant. Similarly, Griffin et al*.* and Juhakoski et al*.* highlight dominance of the physiotherapeutic treatment [42, 56]. However, Tan et al*.* found, that the physiotherapeutic intervention involved cost savings but was also less effective [89] (Figs. 4, 5).

The 19 knee complaint clinical trials bear 20 included articles. Two articles built on one clinical trial dealing with chronic knee pain [64, 65]. One article deals with patellofemoral pain [89], one with ACL tears [35], two with meniscal tears [90, 92], and two with knee pain [20, 54]. The remaining twelve articles investigated treatments for knee osteoarthritis. Patients participated in 1 to 18 sessions spread out mostly over 12 weeks (minimum 6 weeks, maximum 12 months). Inpatients were treated up to twice a day and all patients were treated at least once a month. The intervention for knee pain patients lasted the longest—24 months. Interventions contained mainly individual as well as group-based exercise therapy and education, but manual therapy was also offered in some clinical trials. The control groups received usual care, injections, surgery, education, and physiotherapeutic treatments (Table 2). In total the articles include 40 treatment comparisons (Figs. 4, 5).

Of the five trials on mixed lower limb conditions, we included five articles dealing with hip and knee osteoarthritis [13, 25, 31, 36, 74], and one article on ankle fractures [66]. The patients participated in 6 to 18 sessions spread out over 3 to 12 weeks. Inpatients again received treatments up to twice a day, but most interventions were offered 1–2 times a week. The sessions lasted between 50 and 75 min and contained individual physiotherapeutic treatments, manual therapy, and individual and group-based exercises. The control groups received information, usual care, and individual physiotherapeutic treatments (Table 2). Three articles favored a physiotherapeutic treatment. The remaining articles compared different physiotherapeutic treatments with each other. It was highlighted by the authors, that exercise therapy (individual and group) and education were more cost-effective than usual physiotherapeutic care [25], and that individual physiotherapy alone is cost-effective compared to individual physiotherapy combined with manual therapy [66] (Figs. 4, 5).

#### Other Conditions

Other conditions evaluated in cost-effectiveness studies included the complex regional pain syndrome [19], musculoskeletal problems [33], rheumatoid arthritis [91], and mixed patients [44, 65, 79] consisting of older adults, sedentary adults, and orthopedic outpatients. Patients participated in 5 to 60 sessions over a maximum of 24 months. The interventions contained individual pain exposure therapy, physiotherapeutic care, education, manual therapy, and group exercises. The control groups received usual care and exercises (Table 2). In each of the six articles only one cost-effectiveness comparison was performed (Figs. 4, 5).

#### Summary

The provided details on the delivered treatments varied widely between articles. A type of treatment was mentioned in all, however three articles only named “physiotherapy” as treatment without further explanations. Information on the frequency of the treatment was given in 67 articles; 16 provided no information. Similarly, the intensity was mentioned in 53 articles and missing in 30 articles. However, 28 of the 53 articles providing information on the intensity only mention that it was increased during the intervention or tailored to the patient without further explanation. Finally, the dose was mentioned in 55 and lacking in 28 articles (Table 2).

Due to the limited description of the interventions and the heterogeneity of treatment combinations in the intervention and control groups, summarizing comparisons based on interventions was largely restricted.

Nonetheless, in the 81 comparisons between a physiotherapeutic intervention and any other intervention we found the following insights [13, 14, 19, 20, 23, 26, 29, 30, 33–35, 37, 39, 41, 42, 45–49, 51–53, 55–57, 59, 61–63, 65, 67, 68, 72, 74, 76, 79–82, 84–92, 95–97]: 27 comparisons were performed between a physiotherapeutic intervention (without care provided by a medical doctor) and an intervention involving care from a medical doctor (Fig. 4) [13, 23, 26, 33, 34, 41, 46, 47, 49, 51, 52, 56, 62, 65, 72–74, 88, 91]. Of those about 50% (n = 13) were dominant [13, 34, 46, 47, 49, 51, 56, 62, 72, 74], two were dominated [52, 91], one involved lower costs and lower effects [88], and 11 had better outcomes but involved higher costs [13, 23, 26, 33, 41, 52, 74, 96]. Seven comparisons involved surgery in the control group [35, 37, 42, 85, 92], most of these (n = 6) were more expensive than the physiotherapeutic intervention [35, 37, 42, 85, 92]. The clinical outcome of the physiotherapeutic interventions was better in three of the comparisons [37, 42, 92]. Additionally, we found three comparisons between an intervention provided by a physiotherapist with one provided by a chiropractor [29, 81, 82]. Two originate from the same study and found physiotherapeutic care to be dominant, however, in the third comparison physiotherapeutic care was dominated. Injections were compared to solely other treatments in five comparisons stemming from three studies [30, 48, 53]. In three of these comparisons, physiotherapeutic care was more expensive but led also to better effects [30, 53]. In the remaining two comparisons (from the same study) physiotherapeutic care was cheaper but also less effective (Fig. 4).

Among the 69 comparisons between two physiotherapeutic interventions [13–18, 21, 22, 24–28, 31, 32, 38, 40, 43, 44, 48, 50, 52, 54, 57, 58, 60, 62–64, 66, 69–71, 74, 75, 77, 78, 80, 83, 86, 93, 94], we found 57 mentioning exercises as a treatment modality [13–18, 21, 22, 25–28, 31, 32, 48, 54, 57, 58, 60, 63, 64, 69–71, 74, 75, 78, 80, 83, 94, 93], however, it should be noted that if usual physiotherapy was provided this could also include exercises. Nonetheless, since it was not explicitly mentioned we do not consider “usual physiotherapy” as any intervention involving exercises. Of those comparisons 22 mentioned exercises for both comparators. Twenty-four of the comparisons involved manual therapy as part of an intervention, four in both groups [13, 16–18, 24, 26, 62–64, 66, 74, 75, 94]. Again, it should be noted that individual physiotherapy could have included manual therapies as well but this was not mentioned. Overall, there was no clear trend regarding the cost and the clinical outcomes observed. Having a closer look at comparisons between group-based and individual physiotherapeutic interventions, we found no clear trend regarding cost-effectiveness. This can partly be explained by the mixed treatments that were involved in these interventions as well, hindering clear comparisons.

## Discussion

### Key Results

Several good quality cost-effectiveness evaluations of physiotherapeutic interventions for patients with musculoskeletal conditions exist. Low back and knee conditions are frequently evaluated, however, for conditions addressing other joints none to few studies are available, here further research is needed. Unfortunately, the description of investigated interventions is often limited in detail and the combination of treatments varies widely. This restricted the ability to fairly compare different treatments.

In the comparisons between a physiotherapeutic intervention and those provided by other health professionals, a minor indication of physiotherapy was found to be cost-effective. Of the 42 comparisons between physiotherapeutic care and care provided by a chiropractor or a medical doctor involving surgeries and injections, we found that 18 were dominant and only four were dominated. For the 14 comparisons with higher costs and better effects, as well as for the 6 with a lower effect and lower costs the willingness to pay is crucial for deciding if the treatment should be considered cost-effective or not. The identification of which physiotherapeutic interventions are cost-effective was hindered by clear descriptions of the provided interventions and similar comparisons of treatment combinations.

To the best of our knowledge, this is the first review summarizing the findings of cost-effectiveness evaluations of physiotherapeutic interventions for musculoskeletal conditions. The earliest full-economic evaluation of a physiotherapeutic intervention for a musculoskeletal condition was published in 1994. However, since 2003 at least one new trial-based economic evaluation article has been released each year. This long inclusion period should be noticed when evaluating the relevance of a specific recommended treatment. The physiotherapeutic care and also the comparison treatments could have been enhanced during this time, meaning careful consideration of the (albeit often limited) intervention details is necessary before implementation.

Similarly, when utilizing and comparing the individual study findings of this review the underlying context of the study, the clinical outcome measures, and the findings of the Health Economic Criteria checklist should be considered. Different healthcare systems, as well as the culture of clinicians and patients could influence the cost-effectiveness. As an example, while in some countries like the Netherlands, Great Britain, and Sweden the patients may proceed directly to the physiotherapist, in other countries like Germany they have to consult a medical doctor first—this of course affects the overall cost-effectiveness and should be considered when studies from different countries are compared. Similarly, summarizing and comparing studies that utilized different health outcome measures is challenging. Of course, it is most important that the clinical outcomes are relevant to the patient, however, if different health outcome measures are utilized the comparison of the studies is limited, as the cost-effectiveness might change with a different health outcome. Finally, attention should be given to the evaluation of the Health Economic Criteria checklist of the individual studies/comparisons, which we do not indicate in the cost-effectiveness plane.

The availability of economic evaluation studies in our work mirrors the prevalence of the conditions, which is in line with previous reviews [9, 98]. We found that osteoarthritis was frequently studied. Similarly, a review of orthopedic surgery interventions found that joint arthroplasty, which is the last treatment option for patients with hip and knee osteoarthritis, was commonly investigated in related cost-effectiveness analyses [98]. Additionally, like our work, a review on physical exercise found most cost-effectiveness studies involved back conditions, osteoarthritis, and knee pain [10]. Single studies were also found for the musculoskeletal disorders of the shoulder and the neck [10].

That review [10] on physical exercise in the treatment of various diseases overlaps partly with our study. It includes 28 studies on musculoskeletal conditions of which we included 12 as well. However, the investigators focused on exercises as an intervention while we focused on treatments delivered by physiotherapists.

Interestingly, exercise was the most studied physiotherapeutic treatment in our work. Unfortunately, the treatments like exercise considered in our review were often combined with other treatments such as education, which often leaves the effectiveness of one specific treatment modality open. Furthermore, the treatments were rarely described sufficiently. For exercises e.g. they often lacked at least one dimension of frequency, intensity, time, and type of exercises e.g. aerobic vs. anaerobic or group vs individual therapy. Some articles even mentioned physiotherapy as provided treatment only. However, physiotherapy is not a treatment but a profession [99]. Consequently, several of the described interventions lack details, which leaves practitioners with the intention to implement cost-effective treatments with uncertainty and limits the ability to compare between different physiotherapeutic treatments.

The lack of provided details of the provided physiotherapeutic intervention is present in several studies and in clinical practice. Some initiatives aim to improve the documentation of provided treatments [100, 101]. However, they often apply to one specific treatment only such as exercises or the McKenzie treatment method [102]. Therefore, their applicability to other physiotherapeutic treatments is limited and therapists without special training sometimes do not understand the documentation. Hence, a specific but detailed documentation system for the provision of physiotherapeutic treatments is yet to the best of our knowledge missing.

Interestingly the provided treatments were similar across different body parts. This might suggest that the ideal treatment for a painful joint does not depend on the location. However, studies with more detailed treatment descriptions and with mixed population groups need to prove this hypothesis.

### Future Research

The findings of our study indicate that there is a lack of economic evaluations for musculoskeletal conditions affecting joints other than the back and knee. Furthermore, conditions other than osteoarthritis such as fractures were rarely investigated and need further attention. Finally, systematic reviews of economic evaluations for physiotherapeutic treatments of back and knee complaints may be indicated, if not already covered by available reviews [103–106]. Additionally, a systematic review of the cost-effectiveness of preventive interventions could be interesting, since our focus was on studies dealing with patients with musculoskeletal conditions, thus excluding primary preventions.

### Strength and Limitations

The major limitation of our study relates to the investigated interventions. First, defining the inclusion and exclusion criteria for an intervention was challenging. We aimed to include physiotherapeutic interventions, however, there is a large body of treatments such as tai chi, yoga, behavior change techniques, etc. which can be offered by physiotherapists but require further training. This training is open to non-health professionals as well. Some treatments e.g. behavior change techniques can also be offered by other health professionals who may be better qualified and thus more likely to provide a specific treatment. Furthermore, we decided to take a rather narrow approach regarding the inclusion criteria to ensure that the intervention was offered by physiotherapists. Second, we excluded conference abstracts, reviews, and model-based studies, which might involve the exclusion of relevant publications. Finally, our systematic search could have been broadened by additional search terms and the involvement of an additional database. This could have identified additional relevant publications since two of the included publications were only identified through the recommendation of experts in the field. The major strength of this overview is its transparency. Besides publishing a protocol and providing details on the conduct of our study and using the PRISMA statement, we also provide a list of the excluded full-text publications. The qualitative completeness of this review is underpinned by the quality evaluation of the individual articles and the provision of a cost-effectiveness plane. Consequently, we provide insights on the availability and quality of available articles and in this way highlight knowledge gaps in the literature.

### Conclusions

Several high-quality trial-based economic evaluations of physiotherapeutic interventions for patients with musculoskeletal disorders exist and demonstrate cost-effectiveness. However, most articles address low back and knee conditions, and evaluations concerning other joints are limited. Finally, the documentation of provided interventions needs improvement to enable clinicians and stakeholders to fairly compare and finally to implement cost-effective treatments.

### Supplementary Information

**Additional file 1.** Details on the PubMed search.

**Additional file 2.** Overview of excluded full text articles.

**Additional file 3.** Evaluation of the study quality with the Consensus on Health Economic Criteria checklist.

## Data Availability

All data involved data in this article are available online through the original articles. Where there is uncertainty, the authors are happy to provide details on the analyses on request.

## Abbreviations

QALY: Quality-adjusted life-year
RCT: Randomized controlled trial
