# Supplementary material for: Cost-Effectiveness of Treatments for Musculoskeletal Conditions Offered by Physiotherapists: A Systematic Review of Trial-Based Evaluations
Source: Sports Med Open. 2024 Apr 13;10:38. doi: 10.1186/s40798-024-00713-9 (PMC11016054; doi:10.1186/s40798-024-00713-9)
Supplement: Supplementary file 1 — Additional file 1. Details on the PubMed search. [file 40798_2024_713_MOESM1_ESM.docx]

Table S1. Details on the PubMed search

Search: **((((economic analy*) OR (cost analy*) OR (cost benefit*) OR (cost utility) OR (cost effectiveness) OR (economic evaluation)))**

**AND (((muskuloskeletal) OR (chirur*) OR (orthop*) OR (osteoarthritis) OR (back pain))))**

**AND (((orthopaedic rehabilitation) OR (physical rehabilitation) OR (exercise therap*) OR (conservative therap*) OR (conservative management) OR (conservative treatment) OR (exercise training) OR (physiotherap*)))**

((("economical"[All Fields] OR "economics"[MeSH Terms] OR "economics"[All Fields] OR "economic"[All Fields] OR "economically"[All Fields] OR "economics"[MeSH Subheading] OR "economization"[All Fields] OR "economize"[All Fields] OR "economized"[All Fields] OR "economizes"[All Fields] OR "economizing"[All Fields]) AND "analy*"[All Fields]) OR (("economics"[MeSH Subheading] OR "economics"[All Fields] OR "cost"[All Fields] OR "costs and cost analysis"[MeSH Terms] OR ("costs"[All Fields] AND "cost"[All Fields] AND "analysis"[All Fields]) OR "costs and cost analysis"[All Fields]) AND "analy*"[All Fields]) OR (("economics"[MeSH Subheading] OR "economics"[All Fields] OR "cost"[All Fields] OR "costs and cost analysis"[MeSH Terms] OR ("costs"[All Fields] AND "cost"[All Fields] AND "analysis"[All Fields]) OR "costs and cost analysis"[All Fields]) AND "benefit*"[All Fields]) OR (("economics"[MeSH Subheading] OR "economics"[All Fields] OR "cost"[All Fields] OR "costs and cost analysis"[MeSH Terms] OR ("costs"[All Fields] AND "cost"[All Fields] AND "analysis"[All Fields]) OR "costs and cost analysis"[All Fields]) AND ("statistics and numerical data"[MeSH Subheading] OR ("statistics"[All Fields] AND "numerical"[All Fields] AND "data"[All Fields]) OR "statistics and numerical data"[All Fields] OR "utilization"[All Fields] OR "utilisation"[All Fields] OR "utilisations"[All Fields] OR "utilise"[All Fields] OR "utilised"[All Fields] OR "utilises"[All Fields] OR "utilising"[All Fields] OR "utilities"[All Fields] OR "utility"[All Fields] OR "utilizations"[All Fields] OR "utilize"[All Fields] OR "utilized"[All Fields] OR "utilizer"[All Fields] OR "utilizers"[All Fields] OR "utilizes"[All Fields] OR "utilizing"[All Fields])) OR ("cost benefit analysis"[MeSH Terms] OR ("cost benefit"[All Fields] AND "analysis"[All Fields]) OR "cost benefit analysis"[All Fields] OR ("cost"[All Fields] AND "effectiveness"[All Fields]) OR "cost effectiveness"[All Fields]) OR ("cost benefit analysis"[MeSH Terms] OR ("cost benefit"[All Fields] AND "analysis"[All Fields]) OR "cost benefit analysis"[All Fields] OR ("economic"[All Fields] AND "evaluation"[All Fields]) OR "economic evaluation"[All Fields])) AND ("muskuloskeletal"[All Fields] OR "chirur*"[All Fields] OR "orthop*"[All Fields] OR ("osteoarthritis"[MeSH Terms] OR "osteoarthritis"[All Fields] OR "osteoarthritides"[All Fields]) OR ("back pain"[MeSH Terms] OR ("back"[All Fields] AND "pain"[All Fields]) OR "back pain"[All Fields])) AND ((("orthopaedic"[All Fields] OR "orthopedics"[MeSH Terms] OR "orthopedics"[All Fields] OR "orthopedic"[All Fields] OR "orthopaedical"[All Fields] OR "orthopedical"[All Fields] OR "orthopaedics"[All Fields]) AND ("rehabilitant"[All Fields] OR "rehabilitants"[All Fields] OR "rehabilitate"[All Fields] OR "rehabilitated"[All Fields] OR "rehabilitates"[All Fields] OR "rehabilitating"[All Fields] OR "rehabilitation"[MeSH Terms] OR "rehabilitation"[All Fields] OR "rehabilitations"[All Fields] OR "rehabilitative"[All Fields] OR "rehabilitation"[MeSH Subheading] OR "rehabilitation s"[All Fields] OR "rehabilitational"[All Fields] OR "rehabilitator"[All Fields] OR "rehabilitators"[All Fields])) OR (("physical examination"[MeSH Terms] OR ("physical"[All Fields] AND "examination"[All Fields]) OR "physical examination"[All Fields] OR "physical"[All Fields] OR "physically"[All Fields] OR "physicals"[All Fields]) AND ("rehabilitant"[All Fields] OR "rehabilitants"[All Fields] OR "rehabilitate"[All Fields] OR "rehabilitated"[All Fields] OR "rehabilitates"[All Fields] OR "rehabilitating"[All Fields] OR "rehabilitation"[MeSH Terms] OR "rehabilitation"[All Fields] OR "rehabilitations"[All Fields] OR "rehabilitative"[All Fields] OR "rehabilitation"[MeSH Subheading] OR "rehabilitation s"[All Fields] OR "rehabilitational"[All Fields] OR "rehabilitator"[All Fields] OR "rehabilitators"[All Fields])) OR (("exercise"[MeSH Terms] OR "exercise"[All Fields] OR "exercises"[All Fields] OR "exercise therapy"[MeSH Terms] OR ("exercise"[All Fields] AND "therapy"[All Fields]) OR "exercise therapy"[All Fields] OR "exercise s"[All Fields] OR "exercised"[All Fields] OR "exerciser"[All Fields] OR "exercisers"[All Fields] OR "exercising"[All Fields]) AND "therap*"[All Fields]) OR (("conservancies"[All Fields] OR "conservancy"[All Fields] OR "conservancy s"[All Fields] OR "conservation"[All Fields] OR "conservational"[All Fields] OR "conservations"[All Fields] OR "conservative"[All Fields] OR "conservatively"[All Fields] OR "conservatives"[All Fields] OR "conserve"[All Fields] OR "conserved"[All Fields] OR "conserves"[All Fields] OR "conserving"[All Fields]) AND "therap*"[All Fields]) OR ("conservative treatment"[MeSH Terms] OR ("conservative"[All Fields] AND "treatment"[All Fields]) OR "conservative treatment"[All Fields] OR ("conservative"[All Fields] AND "management"[All Fields]) OR "conservative management"[All Fields]) OR ("conservative treatment"[MeSH Terms] OR ("conservative"[All Fields] AND "treatment"[All Fields]) OR "conservative treatment"[All Fields]) OR ("exercise"[MeSH Terms] OR "exercise"[All Fields] OR ("exercise"[All Fields] AND "training"[All Fields]) OR "exercise training"[All Fields]) OR "physiotherap*"[All Fields])

**Translations**

**economic:** "economical"[All Fields] OR "economics"[MeSH Terms] OR "economics"[All Fields] OR "economic"[All Fields] OR "economically"[All Fields] OR "economics"[Subheading] OR "economization"[All Fields] OR "economize"[All Fields] OR "economized"[All Fields] OR "economizes"[All Fields] OR "economizing"[All Fields]

**cost:** "economics"[Subheading] OR "economics"[All Fields] OR "cost"[All Fields] OR "costs and cost analysis"[MeSH Terms] OR ("costs"[All Fields] AND "cost"[All Fields] AND "analysis"[All Fields]) OR "costs and cost analysis"[All Fields]

**cost:** "economics"[Subheading] OR "economics"[All Fields] OR "cost"[All Fields] OR "costs and cost analysis"[MeSH Terms] OR ("costs"[All Fields] AND "cost"[All Fields] AND "analysis"[All Fields]) OR "costs and cost analysis"[All Fields]

**cost:** "economics"[Subheading] OR "economics"[All Fields] OR "cost"[All Fields] OR "costs and cost analysis"[MeSH Terms] OR ("costs"[All Fields] AND "cost"[All Fields] AND "analysis"[All Fields]) OR "costs and cost analysis"[All Fields]

**utility:** "statistics and numerical data"[Subheading] OR ("statistics"[All Fields] AND "numerical"[All Fields] AND "data"[All Fields]) OR "statistics and numerical data"[All Fields] OR "utilization"[All Fields] OR "utilisation"[All Fields] OR "utilisations"[All Fields] OR "utilise"[All Fields] OR "utilised"[All Fields] OR "utilises"[All Fields] OR "utilising"[All Fields] OR "utilities"[All Fields] OR "utility"[All Fields] OR "utilizations"[All Fields] OR "utilize"[All Fields] OR "utilized"[All Fields] OR "utilizer"[All Fields] OR "utilizers"[All Fields] OR "utilizes"[All Fields] OR "utilizing"[All Fields]

**cost effectiveness:** "cost-benefit analysis"[MeSH Terms] OR ("cost-benefit"[All Fields] AND "analysis"[All Fields]) OR "cost-benefit analysis"[All Fields] OR ("cost"[All Fields] AND "effectiveness"[All Fields]) OR "cost effectiveness"[All Fields]

**economic evaluation:** "cost-benefit analysis"[MeSH Terms] OR ("cost-benefit"[All Fields] AND "analysis"[All Fields]) OR "cost-benefit analysis"[All Fields] OR ("economic"[All Fields] AND "evaluation"[All Fields]) OR "economic evaluation"[All Fields]

**osteoarthritis:** "osteoarthritis"[MeSH Terms] OR "osteoarthritis"[All Fields] OR "osteoarthritides"[All Fields]

**back pain:** "back pain"[MeSH Terms] OR ("back"[All Fields] AND "pain"[All Fields]) OR "back pain"[All Fields]

**orthopaedic:** "orthopaedic"[All Fields] OR "orthopedics"[MeSH Terms] OR "orthopedics"[All Fields] OR "orthopedic"[All Fields] OR "orthopaedical"[All Fields] OR "orthopedical"[All Fields] OR "orthopaedics"[All Fields]

**rehabilitation:** "rehabilitant"[All Fields] OR "rehabilitant's"[All Fields] OR "rehabilitants"[All Fields] OR "rehabilitate"[All Fields] OR "rehabilitated"[All Fields] OR "rehabilitates"[All Fields] OR "rehabilitating"[All Fields] OR "rehabilitation"[MeSH Terms] OR "rehabilitation"[All Fields] OR "rehabilitations"[All Fields] OR "rehabilitative"[All Fields] OR "rehabilitation"[Subheading] OR "rehabilitation's"[All Fields] OR "rehabilitational"[All Fields] OR "rehabilitator"[All Fields] OR "rehabilitators"[All Fields]

**physical:** "physical examination"[MeSH Terms] OR ("physical"[All Fields] AND "examination"[All Fields]) OR "physical examination"[All Fields] OR "physical"[All Fields] OR "physically"[All Fields] OR "physicals"[All Fields]

**rehabilitation:** "rehabilitant"[All Fields] OR "rehabilitant's"[All Fields] OR "rehabilitants"[All Fields] OR "rehabilitate"[All Fields] OR "rehabilitated"[All Fields] OR "rehabilitates"[All Fields] OR "rehabilitating"[All Fields] OR "rehabilitation"[MeSH Terms] OR "rehabilitation"[All Fields] OR "rehabilitations"[All Fields] OR "rehabilitative"[All Fields] OR "rehabilitation"[Subheading] OR "rehabilitation's"[All Fields] OR "rehabilitational"[All Fields] OR "rehabilitator"[All Fields] OR "rehabilitators"[All Fields]

**exercise:** "exercise"[MeSH Terms] OR "exercise"[All Fields] OR "exercises"[All Fields] OR "exercise therapy"[MeSH Terms] OR ("exercise"[All Fields] AND "therapy"[All Fields]) OR "exercise therapy"[All Fields] OR "exercise's"[All Fields] OR "exercised"[All Fields] OR "exerciser"[All Fields] OR "exercisers"[All Fields] OR "exercising"[All Fields]

**conservative:** "conservancies"[All Fields] OR "conservancy"[All Fields] OR "conservancy's"[All Fields] OR "conservation"[All Fields] OR "conservational"[All Fields] OR "conservations"[All Fields] OR "conservative"[All Fields] OR "conservatively"[All Fields] OR "conservatives"[All Fields] OR "conserve"[All Fields] OR "conserved"[All Fields] OR "conserves"[All Fields] OR "conserving"[All Fields]

**conservative management:** "conservative treatment"[MeSH Terms] OR ("conservative"[All Fields] AND "treatment"[All Fields]) OR "conservative treatment"[All Fields] OR ("conservative"[All Fields] AND "management"[All Fields]) OR "conservative management"[All Fields]

**conservative treatment:** "conservative treatment"[MeSH Terms] OR ("conservative"[All Fields] AND "treatment"[All Fields]) OR "conservative treatment"[All Fields]

**exercise training:** "exercise"[MeSH Terms] OR "exercise"[All Fields] OR ("exercise"[All Fields] AND "training"[All Fields]) OR "exercise training"[All Fields]
