# Supplementary material for: Cost-Effectiveness of Treatments for Musculoskeletal Conditions Offered by Physiotherapists: A Systematic Review of Trial-Based Evaluations
Source: Sports Med Open. 2024 Apr 13;10:38. doi: 10.1186/s40798-024-00713-9 (PMC11016054; doi:10.1186/s40798-024-00713-9)
Supplement: Supplementary file 2 — Additional file 2. Overview of excluded full text articles. [file 40798_2024_713_MOESM2_ESM.docx]

Table S2. Overview of excluded full text articles

| **Reason for exclusion** | **Records excluded** |
| --- | --- |
| **Intervention not matching, n = 74** |  |
| No (pure) PT intervention, n = 72 | - Aichmair, A.; Burgstaller, J. M.; Schwenkglenks, M.; Steurer, J.; Porchet, F.; Brunner, F.; Farshad, M. (2017): Cost-effectiveness of conservative versus surgical treatment strategies of lumbar spinal stenosis in the Swiss setting: analysis of the prospective multicenter Lumbar Stenosis Outcome Study (LSOS). In: *Eur Spine J* 26 (2), S. 501–509. DOI: 10.1007/s00586-016-4937-y. |
|  | - Aras, E. L.; Bunger, C.; Hansen, E. S.; Søgaard, R. (2016): Cost-Effectiveness of Surgical Versus Conservative Treatment for Thoracolumbar Burst Fractures. In: *Spine (Phila Pa 1976)* 41 (4), S. 337–343. DOI: 10.1097/brs.0000000000001219. |
|  | - Barker, K. L.; Room, J.; Knight, R.; Dutton, S. J.; Toye, F.; Leal, J. et al. (2020): Outpatient physiotherapy versus home-based rehabilitation for patients at risk of poor outcomes after knee arthroplasty: CORKA RCT. In: *Health Technol Assess* 24 (65), S. 1–116. DOI: 10.3310/hta24650. |
|  | - Baulig C, Grams M, Rohrig B, Linck-Eleftheriadis S, Krum menauer F (2015) Clinical outcome and cost effectiveness of inpatient rehabilitation after total hip and knee arthroplasty. A multi-centre cohort benchmarking study between nine rehabilitation departments in Rhineland-Palatinate (Western Germany). Eur J PhysRehabil Med 51(6):803–813 |
|  | - Brealey, S.; Northgraves, M.; Kottam, L.; Keding, A.; Corbacho, B.; Goodchild, L. et al. (2020): Surgical treatments compared with early structured physiotherapy in secondary care for adults with primary frozen shoulder: the UK FROST three-arm RCT. In: *Health Technol Assess* 24 (71), S. 1–162. DOI: 10.3310/hta24710. |
|  | - Bruce, J.; Hossain, A.; Lall, R.; Withers, E. J.; Finnegan, S.; Underwood, M. et al. (2021): Fall prevention interventions in primary care to reduce fractures and falls in people aged 70 years and over: the PreFIT three-arm cluster RCT. In: *Health Technol Assess* 25 (34), S. 1–114. DOI: 10.3310/hta25340. |
|  | - Bührlen, B.; Jäckel, W. H. (2002): [Outpatient orthopaedic rehabilitation: treatment, outcomes and costs as compared to inpatient rehabilitation]. In: *Rehabilitation (Stuttg)* 41 (2-3), S. 148–159. DOI: 10.1055/s-2002-28435. |
|  | - Cochrane, T.; Davey, R. C.; Matthes Edwards, S. M. (2005): Randomised controlled trial of the cost-effectiveness of water-based therapy for lower limb osteoarthritis. In: *Health Technol Assess* 9 (31), iii-iv, ix-xi, 1-114. DOI: 10.3310/hta9310. |
| (cont.) No (pure) PT intervention,  n = 723 | - Comans, T.; Raymer, M.; O'Leary, S.; Smith, D.; Scuffham, P. (2014): Cost-effectiveness of a physiotherapist-led service for orthopaedic outpatients. In: *J Health Serv Res Policy* 19 (4), S. 216–223. DOI: 10.1177/1355819614533675. |
|  | - Corbacho, B.; Brealey, S.; Keding, A.; Richardson, G.; Torgerson, D.; Hewitt, C. et al. (2021): Cost-effectiveness of surgical treatments compared with early structured physiotherapy in secondary care for adults with primary frozen shoulder : an economic evaluation of the UK FROST trial. In: *Bone Jt Open* 2 (8), S. 685–695. DOI: 10.1302/2633-1462.28.Bjo-2021-0075.R1. |
|  | - Crotty, M.; Killington, M.; Liu, E.; Cameron, I. D.; Kurrle, S.; Kaambwa, B. et al. (2019): Should we provide outreach rehabilitation to very old people living in Nursing Care Facilities after a hip fracture? A randomised controlled trial. In: *Age Ageing* 48 (3), S. 373–380. DOI: 10.1093/ageing/afz005. |
|  | - Daffner, S. D.; Hymanson, H. J.; Wang, J. C. (2010): Cost and use of conservative management of lumbar disc herniation before surgical discectomy. In: *Spine J* 10 (6), S. 463–468. DOI: 10.1016/j.spinee.2010.02.005. |
|  | - Darlow, B.; Stanley, J.; Dean, S.; Abbott, J. H.; Garrett, S.; Wilson, R. et al. (2019): The Fear Reduction Exercised Early (FREE) approach to management of low back pain in general practice: A pragmatic cluster-randomised controlled trial. In: *PLoS Med* 16 (9), e1002897. DOI: 10.1371/journal.pmed.1002897. |
|  | - Forster, M.; Veerman, J. L.; Barendregt, J. J.; Vos, T. (2011): Cost-effectiveness of diet and exercise interventions to reduce overweight and obesity. In: *Int J Obes (Lond)* 35 (8), S. 1071–1078. DOI: 10.1038/ijo.2010.246. - Foster, Nadine E.; Dunn, Kate M.; Protheroe, Joanne; Hill, Jonathan C.; Lewis, Martyn; Saunders, Benjamin et al. (2023 Jun): Stratified primary care for adults with musculoskeletal pain: the STarT MSK research programme including RCTs. National Institute for Health and Care Research. Southampton (UK) (Programme Grants for Applied Research). |
|  | - French, S.D., O’Connor, D.A., Green, S.E. *et al.* Improving adherence to acute low back pain guideline recommendations with chiropractors and physiotherapists: the ALIGN cluster randomised controlled trial. *Trials* **23**, 142 (2022). https://doi.org/10.1186/s13063-022-06053-x |
|  | - Glennie, R. A.; Urquhart, J. C.; Koto, P.; Rasoulinejad, P.; Taylor, D.; Sequeira, K. et al. (2021): Microdiscectomy Is More Cost-Effective Than a 6-Month Nonsurgical Care Regimen for Chronic Radiculopathy. In: *Clin Orthop Relat Res*. DOI: 10.1097/corr.0000000000002001. |
| (cont.) No (pure) PT intervention, n = 72 | - Goossens, M. E.; Rutten-van Molken, M. P.; Kole-Snijders, A. M.; Vlaeyen, J. W.; van Breukelen, G.; Leidl, R. (1998): Health economic assessment of behavioural rehabilitation in chronic low back pain: a randomised clinical trial. In: *Health Economics* 7, S. 39–51. |
|  | - Henchoz, Y.; Pinget, C.; Wasserfallen, J. B.; Paillex, R.; Goumoëns, P. de; Norberg, M.; Kai-Lik So, A. (2010): Cost-utility analysis of a three-month exercise programme vs usual care following multidisciplinary rehabilitation for chronic low back pain. In: *J Rehabil Med* 42 (9), S. 846–852. DOI: 10.2340/16501977-0610. |
|  | - Hoeijenbos, M.; Bekkering, T.; Lamers, L.; Hendriks, E.; van Tulder, M.; Koopmanschap, M. (2005): Cost-effectiveness of an active implementation strategy for the Dutch physiotherapy guideline for low back pain. In: *Health Policy* 75 (1), S. 85–98. DOI: 10.1016/j.healthpol.2005.02.008. |
|  | - Hollinghurst, S.; Sharp, D.; Ballard, K.; Barnett, J.; Beattie, A.; Evans, M. et al. (2008): Randomised controlled trial of Alexander technique lessons, exercise, and massage (ATEAM) for chronic and recurrent back pain: economic evaluation. In: *Bmj* 337, a2656. DOI: 10.1136/bmj.a2656. |
|  | - Jain, A.; Marks, M. C.; Kelly, M. P.; Lenke, L. G.; Errico, T. J.; Lonner, B. S. et al. (2019): Cost-Utility Analysis of Operative Versus Nonoperative Treatment of Thoracic Adolescent Idiopathic Scoliosis. In: *Spine (Phila Pa 1976)* 44 (5), S. 309–317. DOI: 10.1097/brs.0000000000002936. |
|  | - Javanbakht, M., Mashayekhi, A., Carlson, A. *et al.* Cost-Effectiveness Analysis of a Medial Meniscus Replacement Prosthesis for the Treatment of Patients with Medial Compartment Pain in the United Kingdom. *PharmacoEconomics Open* **6**, 681–696 (2022). https://doi.org/10.1007/s41669-022-00336-4 |
| (cont.) No (pure) PT intervention, n = 72 | - Jensen, I. B.; Busch, H.; Bodin, L.; Hagberg, J.; Nygren, Å.; Bergström, G. (2009): Cost effectiveness of two rehabilitation programmes for neck and back pain patients: A seven year follow-up. In: *Pain* 142 (3), S. 202–208. DOI: 10.1016/j.pain.2008.12.015. - Kent, Peter; Haines, Terry; O'Sullivan, Peter; Smith, Anne; Campbell, Amity; Schutze, Robert et al. (2023): Cognitive functional therapy with or without movement sensor biofeedback versus usual care for chronic, disabling low back pain (RESTORE): a randomised, controlled, three-arm, parallel group, phase 3, clinical trial. In: Lancet (London, England) 401 (10391), S. 1866–1877. DOI: 10.1016/S0140-6736(23)00441-5. - Kolu, Päivi; Suni, Jaana H.; Tokola, Kari; Raitanen, Jani; Rinne, Marjo; Taulaniemi, Annika et al. (2023): Neuromuscular exercise and counseling for treating recurrent low back pain in female healthcare workers-Findings from a 24-month follow-up study of a randomized controlled trial. In: Scandinavian journal of medicine & science in sports 33 (11), S. 2239–2249. DOI: 10.1111/sms.14451. |
|  | - Kominski, G. F.; Heslin, K. C.; Morgenstern, H.; Hurwitz, E. L.; Harber, P. I. (2005): Economic evaluation of four treatments for low-back pain: results from a randomized controlled trial. In: *Med Care* 43 (5), S. 428–435. DOI: 10.1097/01.mlr.0000160379.12806.08. |
|  | - Konstantinou, K.; Lewis, M.; Dunn, K. M.; Ogollah, R.; Artus, M.; Hill, J. C. et al. (2020): Stratified care versus usual care for management of patients presenting with sciatica in primary care (SCOPiC): a randomised controlled trial. In: *Lancet Rheumatol* 2 (7), e401-e411. DOI: 10.1016/s2665-9913(20)30099-0. |
|  | - Lamb, S. E.; Hansen, Z.; Lall, R.; Castelnuovo, E.; Withers, E. J.; Nichols, V. et al. (2010): Group cognitive behavioural treatment for low-back pain in primary care: a randomised controlled trial and cost-effectiveness analysis. In: *Lancet* 375 (9718), S. 916–923. DOI: 10.1016/s0140-6736(09)62164-4. |
|  | - Leung, K. L.; Lau, T. W.; Chan, H. W.; Chiu, K. C.; Fang, C. X.; Lam, Y. H. et al. (2023): Orthogeriatric co-management model to improve outcome and cost-effectiveness of fragility hip fractures: abridged secondary publication. In: Hong Kong medical journal = Xianggang yi xue za zhi 29 Suppl 2 (1), S. 15–17. - Lodhia, P.; Gui, C.; Chandrasekaran, S.; Suarez-Ahedo, C.; Dirschl, D. R.; Domb, B. G. (2016): The Economic Impact of Acetabular Labral Tears: A Cost-effectiveness Analysis Comparing Hip Arthroscopic Surgery and Structured Rehabilitation Alone in Patients Without Osteoarthritis. In: *Am J Sports Med* 44 (7), S. 1771–1780. DOI: 10.1177/0363546516645532. |
|  | - Lorig, K. R.; Mazonson, P. D.; Holman, H. R. (1993): Evidence suggesting that health education for self-management in patients with chronic arthritis has sustained health benefits while reducing health care costs. In: *Arthritis Rheum* 36 (4), S. 439–446. DOI: 10.1002/art.1780360403. |
|  | - Losina, E.; Smith, K. C.; Paltiel, A. D.; Collins, J. E.; Suter, L. G.; Hunter, D. J. et al. (2019): Cost-Effectiveness of Diet and Exercise for Overweight and Obese Patients With Knee Osteoarthritis. In: *Arthritis Care Res (Hoboken)* 71 (7), S. 855–864. DOI: 10.1002/acr.23716. |
| (cont.) No (pure) PT intervention,  n = 72 | - Malmivaara, A.; Hakkinen, U.; Aro, T.; Heinrichs, M. L.; Koskenniemi, L.; Kuosma, E. et al. (1995): The treatment of acute low back pain: bed rest, exercises, or ordinary activity? In: *New England Journal of Medicine* 332 (6), S. 351–355. DOI: 10.1056/nejm199502093320602. |
|  | - Marks, D.; Bisset, L.; Comans, T.; Thomas, M.; Ng, S. K.; O'Leary, S. et al. (2016): Increasing Capacity for the Treatment of Common Musculoskeletal Problems: A Non-Inferiority RCT and Economic Analysis of Corticosteroid Injection for Shoulder Pain Comparing a Physiotherapist and Orthopaedic Surgeon. In: *PLoS One* 11 (9), e0162679. DOI: 10.1371/journal.pone.0162679. |
|  | - Marsh, J. D.; Birmingham, T. B.; Giffin, J. R.; Isaranuwatchai, W.; Hoch, J. S.; Feagan, B. G. et al. (2016): Cost-effectiveness analysis of arthroscopic surgery compared with non-operative management for osteoarthritis of the knee. In: *BMJ Open* 6 (1), e009949. DOI: 10.1136/bmjopen-2015-009949. |
|  | - Miyamoto, G. C.; Franco, K. F. M.; van Dongen, J. M.; Franco, Yrds; Oliveira, N. T. B. de; Amaral, D. D. V. et al. (2018): Different doses of Pilates-based exercise therapy for chronic low back pain: a randomised controlled trial with economic evaluation. In: *Br J Sports Med* 52 (13), S. 859–868. DOI: 10.1136/bjsports-2017-098825. |
|  | - Niehaus, Richard; Schleicher, Alisa; Ammann, Elias; Kriechling, Philipp; Lenz, Christopher G.; Masanneck, Michael et al. (2023): Operative vs. conservative treatment of AC-Joint Dislocations Rockwood grade ≥ III -An economical and clinical evaluation. In: Cost effectiveness and resource allocation : C/E 21 (1), S. 63. DOI: 10.1186/s12962-023-00468-2. - Oppong, R.; Jowett, S.; Nicholls, E.; Whitehurst, D. G.; Hill, S.; Hammond, A. et al. (2015): Joint protection and hand exercises for hand osteoarthritis: an economic evaluation comparing methods for the analysis of factorial trials. In: *Rheumatology (Oxford)* 54 (5), S. 876–883. DOI: 10.1093/rheumatology/keu389. |
|  | - Oppong, R.; Jowett, S.; Lewis, M.; Clarkson, K.; Paskins, Z.; Croft, P. et al. (2018): Cost-effectiveness of a model consultation to support self-management in patients with osteoarthritis. In: *Rheumatology (Oxford)* 57 (6), S. 1056–1063. DOI: 10.1093/rheumatology/key037. - Østerås, Nina; Aas, Eline; Moseng, Tuva; van Bodegom-Vos, Leti; Dziedzic, Krysia; Natvig, Bård et al. (2023): Longer-term quality of care, effectiveness, and cost-effectiveness of implementing a model of care for osteoarthritis: A cluster-randomized controlled trial. In: Osteoarthritis and cartilage. DOI: 10.1016/j.joca.2023.10.003. |
| (cont.) No (pure) PT intervention, n = 72 | - Palola, Vili; Hevonkorpi, Teemu P.; Ponkilainen, Ville T.; Launonen, Antti P.; Mattila, Ville M. (2023): Sick leave length and the costs of operatively and conservatively treated distal radius fractures in the working age population: a retrospective cohort study. In: BMC musculoskeletal disorders 24 (1), S. 842. DOI: 10.1186/s12891-023-06963-0. - Parker, S. L.; Godil, S. S.; Mendenhall, S. K.; Zuckerman, S. L.; Shau, D. N.; McGirt, M. J. (2014): Two-year comprehensive medical management of degenerative lumbar spine disease (lumbar spondylolisthesis, stenosis, or disc herniation): a value analysis of cost, pain, disability, and quality of life: clinical article. In: *J Neurosurg Spine* 21 (2), S. 143–149. DOI: 10.3171/2014.3.Spine1320. |
|  | - Patel A, Buszewicz M, Beecham J, Grifﬁn M, Rait G, Nazareth I. et al. Economic evaluation of arthritis self management in primary care. BMJ 2009;339:b3532. |
|  | - Patrick, D. L.; Ramsey, S. D.; Spencer, A. C.; Kinne, S.; Belza, B.; Topolski, T. D. (2001): Economic evaluation of aquatic exercise for persons with osteoarthritis. In: *Med Care* 39 (5), S. 413–424. DOI: 10.1097/00005650-200105000-00002. |
|  | - Pomerance, J.; Zurakowski, D.; Fine, I. (2009): The cost-effectiveness of nonsurgical versus surgical treatment for carpal tunnel syndrome. In: *J Hand Surg Am* 34 (7), S. 1193–1200. DOI: 10.1016/j.jhsa.2009.04.034. |
|  | - Rantonen, J.; Karppinen, J.; Vehtari, A.; Luoto, S.; Viikari-Juntura, E.; Hupli, M. et al. (2016): Cost-effectiveness of providing patients with information on managing mild low-back symptoms in an occupational health setting. In: *BMC Public Health* 16, S. 316. DOI: 10.1186/s12889-016-2974-4. |
|  | - Rhon, D. I.; Greenlee, T. A.; Fritz, J. M. (2019): The Influence of a Guideline-Concordant Stepped Care Approach on Downstream Health Care Utilization in Patients with Spine and Shoulder Pain. In: *Pain Med* 20 (3), S. 476–485. DOI: 10.1093/pm/pny212. |
|  | - Rolving, N.; Sogaard, R.; Nielsen, C. V.; Christensen, F. B.; Bünger, C.; Oestergaard, L. G. (2016): Preoperative Cognitive-Behavioral Patient Education Versus Standard Care for Lumbar Spinal Fusion Patients: Economic Evaluation Alongside a Randomized Controlled Trial. In: *Spine (Phila Pa 1976)* 41 (1), S. 18–25. DOI: 10.1097/brs.0000000000001254. |
|  | - Saha, S.; Grahn, B.; Gerdtham, U. G.; Stigmar, K.; Holmberg, S.; Jarl, J. (2019): Structured physiotherapy including a work place intervention for patients with neck and/or back pain in primary care: an economic evaluation. In: *Eur J Health Econ* 20 (2), S. 317–327. DOI: 10.1007/s10198-018-1003-1. |
| (cont.) No (pure) PT intervention, n = 72 | - Saito, Tomohiro; Hamada, Junichiro; Sasanuma, Hideyuki; Iijima, Yuki; Saitsu, Akihiro; Minagawa, Hiroshi et al. (2023): Clinical outcomes and cost-effectiveness of manipulation under brachial plexus block versus physiotherapy for refractory frozen shoulder: a prospective observational study. In: JSES international 7 (6), S. 2410–2419. DOI: 10.1016/j.jseint.2023.07.017. - Scott, B. L.; Lee, C. S.; Shi, L. L.; Lee, M. J.; Athiviraham, A. (2020): Nonoperative Management of Hip Labral Tears Yields Similar Total Hip Arthroplasty Conversion Rate to Arthroscopic Treatment. In: *J Arthroplasty* 35 (1), 23-27.e1. DOI: 10.1016/j.arth.2019.08.042. |
|  | - Serikova-Esengeldina, D., Glushkova, N., Abdushukurova, G. *et al.* Cost-utility analysis of total knee arthroplasty alone and in comparison with post-surgical rehabilitation and conservative treatment in the Republic of Kazakhstan. *Cost Eff Resour Alloc* **20**, 47 (2022). https://doi.org/10.1186/s12962-022-00379-8 |
|  | - Shvartzman, L.; Weingarten, E.; Sherry, H.; Levin, S.; Persaud, A. (1992): Cost-effectiveness analysis of extended conservative therapy versus surgical intervention in the management of herniated lumbar intervertebral disc. In: *Spine (Phila Pa 1976)* 17 (2), S. 176–182. DOI: 10.1097/00007632-199202000-00010. |
|  | - Skou, S. T.; Roos, E. M.; Laursen, M.; Arendt-Nielsen, L.; Rasmussen, S.; Simonsen, O. et al. (2020): Cost-effectiveness of 12 weeks of supervised treatment compared to written advice in patients with knee osteoarthritis: a secondary analysis of the 2-year outcome from a randomized trial. In: *Osteoarthritis Cartilage* 28 (7), S. 907–916. DOI: 10.1016/j.joca.2020.03.009. |
|  | - Smith, K. C.; Losina, E.; Messier, S. P.; Hunter, D. J.; Chen, A. T.; Katz, J. N.; Paltiel, A. D. (2020): Budget Impact of Funding an Intensive Diet and Exercise Program for Overweight and Obese Patients With Knee Osteoarthritis. In: *ACR Open Rheumatol* 2 (1), S. 26–36. DOI: 10.1002/acr2.11090. |
|  | - Steenstra, I. A.; Anema, J. R.; van Tulder, M. W.; Bongers, P. M.; Vet, H. C. de; van Mechelen, W. (2006): Economic evaluation of a multi-stage return to work program for workers on sick-leave due to low back pain. In: *J Occup Rehabil* 16 (4), S. 557–578. DOI: 10.1007/s10926-006-9053-0. |
|  | - Su, A. W.; Bogunovic, L.; Johnson, J.; Klein, S.; Matava, M. J.; McCormick, J. et al. (2020): Operative Versus Nonoperative Treatment of Acute Achilles Tendon Ruptures: A Pilot Economic Decision Analysis. In: *Orthop J Sports Med* 8 (3), 2325967120909918. DOI: 10.1177/2325967120909918. |
| (cont.) No (pure) PT intervention, n = 72 | - Swart, E.; Redler, L.; Fabricant, P. D.; Mandelbaum, B. R.; Ahmad, C. S.; Wang, Y. C. (2014): Prevention and screening programs for anterior cruciate ligament injuries in young athletes: a cost-effectiveness analysis. In: *J Bone Joint Surg Am* 96 (9), S. 705–711. DOI: 10.2106/jbjs.M.00560. |
|  | - Takaki, Shu; Miyama, Hiroshi; Iwasaki, Motoki (2023): Cost-effectiveness analysis of intradiscal condoliase injection vs. surgical or conservative treatment for lumbar disc herniation. In: Journal of medical economics 26 (1), S. 233–242. DOI: 10.1080/13696998.2023.2173465. - Tosteson AN, Skinner JS, Tosteson TD, Lurie JD, Andersson GB, Berven S, Grove MR, Hanscom B, Blood EA, Weinstein JN. The cost effectiveness of surgical versus nonoperative treatment for lumbar disc herniation over two years: evidence from the Spine Patient Outcomes Research Trial (SPORT). Spine. 2008;33:2108–2115. |
|  | - Tousignant, M.; Desrosiers, J.; Tourigny, A.; Robichaud, L. (2005): Costs of a home-based rehabilitation program for older adults after lower limb orthopedic surgery: a pilot study. In: *Arch Gerontol Geriatr* 41 (1), S. 51–60. DOI: 10.1016/j.archger.2004.11.002. |
|  | - Truntzer, J. N.; Triana, B.; Harris, A. H. S.; Baker, L.; Chou, L.; Kamal, R. N. (2017): Cost-minimization Analysis of the Management of Acute Achilles Tendon Rupture. In: *J Am Acad Orthop Surg* 25 (6), S. 449–457. DOI: 10.5435/jaaos-d-16-00553. |
|  | - Vagnoni, E.; Biavati, G. R.; Felisatti, M.; Pomidori, L. (2018): Moderating healthcare costs through an assisted physical activity programme. In: *Int J Health Plann Manage* 33 (4), S. 1146–1158. DOI: 10.1002/hpm.2596. |
|  | - van den Hout WB, Peul WC, Koes BW, Brand R, Kievit J, Thomeer RT; Leiden-The Hague Spine Intervention Prognostic Study Group: Prolonged conservative care versus early surgery in patients with sciatica from lumbar disc herniation: cost utility analysis alongside a randomised controlled trial. BMJ. 2008;336:1351–1354. |
|  | - Werner, E. L.; Storheim, K.; Løchting, I.; Wisløff, T.; Grotle, M. (2016): Cognitive Patient Education for Low Back Pain in Primary Care: A Cluster Randomized Controlled Trial and Cost-Effectiveness Analysis. In: *Spine (Phila Pa 1976)* 41 (6), S. 455–462. DOI: 10.1097/brs.0000000000001268. |
|  | - Westin, O.; Svensson, M.; Nilsson Helander, K.; Samuelsson, K.; Grävare Silbernagel, K.; Olsson, N. et al. (2018): Cost-effectiveness analysis of surgical versus non-surgical management of acute Achilles tendon ruptures. In: *Knee Surg Sports Traumatol Arthrosc* 26 (10), S. 3074–3082. DOI: 10.1007/s00167-018-4953-z. |
| (cont.) No (pure) PT intervention, n = 72 | - Whitehurst, D. G.; Bryan, S.; Hay, E. M.; Thomas, E.; Young, J.; Foster, N. E. (2011): Cost-effectiveness of acupuncture care as an adjunct to exercise-based physical therapy for osteoarthritis of the knee. In: *Phys Ther* 91 (5), S. 630–641. DOI: 10.2522/ptj.20100239. |
|  | - Williams, A.; van Dongen, J. M.; Kamper, S. J.; O'Brien, K. M.; Wolfenden, L.; Yoong, S. L. et al. (2019): Economic evaluation of a healthy lifestyle intervention for chronic low back pain: A randomized controlled trial. In: *Eur J Pain* 23 (3), S. 621–634. DOI: 10.1002/ejp.1334. - Wright A; Lloyd-Davies A; Williams S; Ellis R; Strike P; Wright, Alison et al. (2005): Individual active treatment combined with group exercise for acute and subacute low back pain. In: Spine 30 (11), S. 1235–1241. DOI: 10.1097/01.brs.0000164266.00150.b6. |
|  | - Yates, C. J.; Chauchard, M. A.; Liew, D.; Bucknill, A.; Wark, J. D. (2015): Bridging the osteoporosis treatment gap: performance and cost-effectiveness of a fracture liaison service. In: *J Clin Densitom* 18 (2), S. 150–156. DOI: 10.1016/j.jocd.2015.01.003. - Zhou, Quan; He, Wei; Lv, Jiaheng; Liu, Hao; Yang, Huilin; Zhang, Junxin; Liu, Tao (2023): Benefits of Early Surgical Treatment for Patients with Multilevel Cervical Canal Stenosis of Acute Traumatic Central Cord Syndrome. In: Orthopaedic surgery 15 (12), S. 3092–3100. DOI: 10.1111/os.13904. |
| E-Health intervention, n = 2 | - Hunter, David J.; Bowden, Jocelyn L.; Hinman, Rana S.; Egerton, Thorlene; Briggs, Andrew M.; Bunker, Stephen J. et al. (2023): Effectiveness of a New Service Delivery Model for Management of Knee Osteoarthritis in Primary Care: A Cluster Randomized Controlled Trial. In: ARTHRITIS CARE RES (2151464X) 75 (6), S. 1320–1332. DOI: 10.1002/acr.25037. - Kaufman BG, Allen KD, Coffman CJ, Woolson S, Caves K, Hall K, Hoenig HM, Huffman KM, Morey MC, Hodges NJ, Ramasunder S, van Houtven CH. Cost and Quality of Life Outcomes of the STepped Exercise Program for Patients With Knee OsteoArthritis Trial. Value Health. 2022 Apr;25(4):614-621. doi: 10.1016/j.jval.2021.09.018 |
| **Control group not matching, n = 1** |  |
| No control group involved,  n = 1 | - Haas M. Evaluation of physiotherapy using cost-utility analysis. Aust J Physiother. 1993;39(3):211-216. |
| **Outcome not matching, n = 28** |  |
| No cost-(effectiveness) analysis,  n = 20 | - Barker, K. L.; Room, J.; Knight, R.; Dutton, S.; Toye, F.; Leal, J. et al. (2021): Home-based rehabilitation programme compared with traditional physiotherapy for patients at risk of poor outcome after knee arthroplasty: the CORKA randomised controlled trial. In: *BMJ Open* 11 (8), e052598. DOI: 10.1136/bmjopen-2021-052598. |
|  | - Başar, B.; Başar, G.; Büyükkuşçu, M. Ö.; Başar, H. (2021): Comparison of physical therapy and arthroscopic partial meniscectomy treatments in degenerative meniscus tears and the effect of combined hyaluronic acid injection with these treatments: A randomized clinical trial. In: *J Back Musculoskelet Rehabil* 34 (5), S. 767–774. DOI: 10.3233/bmr-200284. |
|  | - Bendix, T.; Bendix, A.; Labriola, M.; Haestrup, C.; Ebbehøj, N. (2000): Functional restoration versus outpatient physical training in chronic low back pain: a randomized comparative study. In: *Spine (Phila Pa 1976)* 25 (19), S. 2494–2500. DOI: 10.1097/00007632-200010010-00012. |
|  | - Brown, K. C.; Sirles, A. T.; Hilyer, J. C.; Thomas, M. J. (1992): Cost-effectiveness of a back school intervention for municipal employees. In: *Spine (Phila Pa 1976)* 17 (10), S. 1224–1228. DOI: 10.1097/00007632-199210000-00016. |
|  | - Brox JI, Staff PH, Ljunggren AE, et al. Arthroscopic surgery compared with supervised exercises in patients with rotator cuff disease (stage II impingement syndrome). BMJ. 1993;307:899-903. |
|  | - Fritz, J. M.; Childs, J. D.; Wainner, R. S.; Flynn, T. W. (2012): Primary care referral of patients with low back pain to physical therapy: impact on future health care utilization and costs. In: *Spine (Phila Pa 1976)* 37 (25), S. 2114–2121. DOI: 10.1097/BRS.0b013e31825d32f5. |
|  | - Fritz, J. M.; Brennan, G. P.; Hunter, S. J.; Magel, J. S. (2013): Initial management decisions after a new consultation for low back pain: implications of the usage of physical therapy for subsequent health care costs and utilization. In: *Arch Phys Med Rehabil* 94 (5), S. 808–816. DOI: 10.1016/j.apmr.2013.01.008. |
|  | - Frogner, B. K.; Harwood, K.; Andrilla, C. H. A.; Schwartz, M.; Pines, J. M. (2018): Physical Therapy as the First Point of Care to Treat Low Back Pain: An Instrumental Variables Approach to Estimate Impact on Opioid Prescription, Health Care Utilization, and Costs. In: *Health Serv Res* 53 (6), S. 4629–4646. DOI: 10.1111/1475-6773.12984. |
|  | - Garrity, B. M.; McDonough, C. M.; Ameli, O.; Rothendler, J. A.; Carey, K. M.; Cabral, H. J. et al. (2020): Unrestricted Direct Access to Physical Therapist Services Is Associated With Lower Health Care Utilization and Costs in Patients With New-Onset Low Back Pain. In: *Phys Ther* 100 (1), S. 107–115. DOI: 10.1093/ptj/pzz152. |
| (cont.) No cost-(effectiveness) analysis, n = 20 | - Horn, M. E.; Fritz, J. M. (2018): Timing of physical therapy consultation on 1-year healthcare utilization and costs in patients seeking care for neck pain: a retrospective cohort. In: *BMC Health Serv Res* 18 (1), S. 887. DOI: 10.1186/s12913-018-3699-0. |
|  | - Jahantiqh, F.; Abdollahimohammad, A.; Firouzkouhi, M.; Ebrahiminejad, V. (2018): Effects of Reiki Versus Physiotherapy on Relieving Lower Back Pain and Improving Activities Daily Living of Patients With Intervertebral Disc Hernia. In: *J Evid Based Integr Med* 23, 2515690x18762745. DOI: 10.1177/2515690x18762745. |
|  | - Lewis, J. S.; Hewitt, J. S.; Billington, L.; Cole, S.; Byng, J.; Karayiannis, S. (2005): A randomized clinical trial comparing two physiotherapy interventions for chronic low back pain. In: *Spine (Phila Pa 1976)* 30 (7), S. 711–721. DOI: 10.1097/01.brs.0000157469.27779.de. - Littlewood, Chris; Moffatt, Maria; Beckhelling, Jacqueline; Davis, Daniel; Burden, Adrian; Pitt, Lisa et al. (2023): Physiotherapist-led exercise versus usual care (waiting-list) control for patients awaiting rotator cuff repair surgery: A pilot randomised controlled trial (POWER). In: Musculoskeletal science & practice 68, S. 102874. DOI: 10.1016/j.msksp.2023.102874. |
|  | - Merkesdal, S.; Mau, W. (2005): Prediction of costs-of-illness in patients with low back pain undergoing orthopedic outpatient rehabilitation. In: *Int J Rehabil Res* 28 (2), S. 119–126. DOI: 10.1097/00004356-200506000-00004. |
|  | - Orndahl, C. M.; Perera, R. A.; Hung, A.; Dumenci, L.; Riddle, D. L. (2021): Physical Therapy Use, Costs, and Value for Latent Classes of Good vs Poor Outcome in Patients Who Catastrophize About Their Pain Prior to Knee Arthroplasty. In: *Arch Phys Med Rehabil* 102 (7), S. 1347–1351. DOI: 10.1016/j.apmr.2021.02.004. |
|  | - Png, M. E.; Griffin, X. L.; Costa, M. L.; Achten, J.; Pinedo-Villanueva, R. (2020): Utilization and costs of formal and informal care, home adaptations, and physiotherapy among older patients with hip fracture. In: *Bone Joint Res* 9 (5), S. 250–257. DOI: 10.1302/2046-3758.95.Bjr-2019-0221.R1. |
|  | - Seferlis, T.; Lindholm, L.; Németh, G. (2000): Cost-minimisation analysis of three conservative treatment programmes in 180 patients sick-listed for acute low-back pain. In: *Scand J Prim Health Care* 18 (1), S. 53–57. DOI: 10.1080/02813430050202578. |
|  | - Then, J. W.; Shivdas, S.; Tunku Ahmad Yahaya, T. S.; Ab Razak, N. I.; Choo, P. T. (2020): Gamification in rehabilitation of metacarpal fracture using cost-effective end-user device: A randomized controlled trial. In: *J Hand Ther* 33 (2), S. 235–242. DOI: 10.1016/j.jht.2020.03.029. |
| (cont.) No cost-(effectiveness) analysis, n = 20 | - Theologis, A. A.; Lau, D.; Dalle-Ore, C.; Tsu, A.; Deviren, V.; Ames, C. P. (2021): Costs and utility of post-discharge acute inpatient rehabilitation following adult spinal deformity surgery. In: *Spine Deform* 9 (3), S. 817–822. DOI: 10.1007/s43390-020-00251-w. |
|  | - Zadro JR, Lewin AM, Kharel P, Naylor J, Maher CG, Harris IA. Physical therapy utilization, costs, and return-to-work status following lumbar spine surgery: A retrospective analysis of workers compensation claims in Australia. Braz J Phys Ther. 2022 Mar-Apr;26(2):100400. doi: 10.1016/j.bjpt.2022.100400 |
| No health outcome, n = 8 |  |
|  | - Childs, J. D.; Fritz, J. M.; Wu, S. S.; Flynn, T. W.; Wainner, R. S.; Robertson, E. K. et al. (2015): Implications of early and guideline adherent physical therapy for low back pain on utilization and costs. In: *BMC Health Serv Res* 15, S. 150. DOI: 10.1186/s12913-015-0830-3. |
|  | - Magel, J.; Kim, J.; Thackeray, A.; Hawley, C.; Petersen, S.; Fritz, J. M. (2018): Associations Between Physical Therapy Continuity of Care and Health Care Utilization and Costs in Patients With Low Back Pain: A Retrospective Cohort Study. In: *Phys Ther* 98 (12), S. 990–999. DOI: 10.1093/ptj/pzy103. |
|  | - Pivec, R.; Stokes, M.; Chitnis, A. S.; Paulino, C. B.; Harwin, S. F.; Mont, M. A. (2013): Clinical and economic impact of TENS in patients with chronic low back pain: analysis of a nationwide database. In: *Orthopedics* 36 (12), S. 922–928. DOI: 10.3928/01477447-20131120-04. |
|  | - Rhon, D. I.; Snodgrass, S. J.; Cleland, J. A.; Greenlee, T. A.; Sissel, C. D.; Cook, C. E. (2018): Comparison of Downstream Health Care Utilization, Costs, and Long-Term Opioid Use: Physical Therapist Management Versus Opioid Therapy Management After Arthroscopic Hip Surgery. In: *Phys Ther* 98 (5), S. 348–356. DOI: 10.1093/ptj/pzy019. |
|  | - Rosenfeld M, Seferiadis A, Gunnarsson R. Active involvement and intervention in patients exposed to whiplash trauma in automobile crashes reduces costs: a randomized, controlled clinical trial and health economic evaluation. Spine. 2006;31(16):1799–1804. - Staunton, Peter F.; Grant-Freemantle, Marc C.; Pomeroy, Eoghan; Cashman, James (2023): The Role of a Seven-Day Physiotherapy Service in Reducing Length of Stay and Improving Cost-Effectiveness in Arthroplasty Surgery. In: Cureus 15 (1), e33951. DOI: 10.7759/cureus.33951. |
| (cont.) No health outcome, n = 8 | - Timm, K. E. (1997): The clinical and cost-effectiveness of two different programs for rehabilitation following ACL reconstruction. In: *J Orthop Sports Phys Ther* 25 (1), S. 43–48. DOI: 10.2519/jospt.1997.25.1.43. |
|  | - Torstensen, T. A.; Ljunggren, A. E.; Meen, H. D.; Odland, E.; Mowinckel, P.; Geijerstam, S. (1998): Efficiency and costs of medical exercise therapy, conventional physiotherapy, and self-exercise in patients with chronic low back pain. A pragmatic, randomized, single-blinded, controlled trial with 1-year follow-up. In: *Spine (Phila Pa 1976)* 23 (23), S. 2616–2624. DOI: 10.1097/00007632-199812010-00017. |
| **Study type not matching, n = 24** |  |
| Systematic review, n = 1 | - Woods, B.; Manca, A.; Weatherly, H.; Saramago, P.; Sideris, E.; Giannopoulou, C. et al. (2017): Cost-effectiveness of adjunct non-pharmacological interventions for osteoarthritis of the knee. In: *PLoS One* 12 (3), e0172749. DOI: 10.1371/journal.pone.0172749. |
| Book, n = 1 | - Zigenfus GC, Yin J, Giang GM, Fogarty WT. Effectiveness of early physical therapy in the treatment of acute low back musculoskeletal disorders. J Occup Environ Med. 2000;42:35-39. |
| Model-based, n = 14 | - Alaia, Erin F.; Subhas, Naveen; Da Silva Cardoso, Madalena; Li, Zachary I.; Shah, Mehul R.; Alaia, Michael J.; Gyftopoulos, Soterios (2023): Common treatment strategies for calcium hydroxyapatite deposition disease: a cost-effectiveness analysis. In: Skeletal Radiol. DOI: 10.1007/s00256-023-04424-2. - Best TM, Petterson S, Plancher K. Sustained acoustic medicine as a non-surgical and non-opioid knee osteoarthritis treatment option: a health economic cost-effectiveness analysis for symptom management. J Orthop Surg Res. 2020;15(1):481. |
|  | - Bove AM, Smith KJ, Bise CG, et al. Exercise, Manual Therapy, and Booster Sessions in Knee Osteoarthritis: Cost-Effectiveness Analysis From a Multicenter Randomized Controlled Trial. Phys Ther. 2018;98(1):16-27. |
|  | - DeFrancesco CJ, Lebrun DG, Molony JT, Jr., Heath MR, Fabricant PD. Safer and Cheaper: An Enhanced Milestone-Based Return to Play Program After Anterior Cruciate Ligament Reconstruction in Young Athletes Is Cost-Effective Compared With Standard Time-Based Return to Play Criteria. Am J Sports Med. 2020;48(5):1100-1107. |
|  | - Farshad M, Gerber C, Meyer DC, Schwab A, Blank PR, Szucs T. Reconstruction versus conservative treatment after rupture of the anterior cruciate ligament: cost effectiveness analysis. BMC Health Serv Res. 2011;11:317. |
| (Cont.) Model-based, n = 14 | - Kang JR, Sin AT, Cheung EV. Treatment of Massive Irreparable Rotator Cuff Tears: A Cost-effectiveness Analysis. Orthopedics. 2017;40(1):e65-e76. - Lilje, Stina; van Tulder, Maurits; Wykman, Anders; Aboagye, Emmanuel; Persson, Ulf (2023): Cost-effectiveness of specialised manual therapy versus orthopaedic care for musculoskeletal disorders: long-term follow-up and health economic model. In: Therapeutic advances in musculoskeletal disease 15, 1759720X221147751. DOI: 10.1177/1759720X221147751. |
|  | - Losina E, Dervan EE, Paltiel AD, et al. Defining the Value of Future Research to Identify the Preferred Treatment of Meniscal Tear in the Presence of Knee Osteoarthritis. PLoS One. 2015;10(6):e0130256. |
|  | - Silva GS, Sullivan JK, Katz JN, Messier SP, Hunter DJ, Losina E. Long-term clinical and economic outcomes of a short-term physical activity program in knee osteoarthritis patients. Osteoarthritis Cartilage. 2020;28(6):735-743. |
|  | - Standfield L, Comans T, Raymer M, O'Leary S, Moretto N, Scuffham P. The Efficiency of Increasing the Capacity of Physiotherapy Screening Clinics or Traditional Medical Services to Address Unmet Demand in Orthopaedic Outpatients: A Practical Application of Discrete Event Simulation with Dynamic Queuing. Appl Health Econ Health Policy. 2016;14(4):479-491. - Stewart, B. A.; Momaya, A. M.; Silverstein, M. D.; Lintner, D. (2017): The Cost-Effectiveness of Anterior Cruciate Ligament Reconstruction in Competitive Athletes. In: Am J Sports Med 45 (1), S. 23–33. DOI: 10.1177/0363546516664719. |
|  | - Teng M, Zhou HJ, Lin L, et al. Cost-effectiveness of hydrotherapy versus land-based therapy in patients with musculoskeletal disorders in Singapore. J Public Health (Oxf). 2019;41(2):391-398. - Vetsch, Thomas; Taeymans, Jan; Lutz, Nathanael (2023): Optimising the current model of care for knee osteoarthritis with the implementation of guideline recommended non-surgical treatments: a model-based health economic evaluation. In: Swiss medical weekly 153, S. 40059. DOI: 10.57187/smw.2023.40059. - Zimmerman, Zoe E.; Cleveland, Rebecca J.; Kostic, Aleksandra M.; Leifer, Valia P.; Weisner, Serena E.; Allen, Kelli D. et al. (2023): Walk with ease for knee osteoarthritis: A cost-effectiveness analysis. In: Osteoarthritis and cartilage open 5 (3), S. 100368. DOI: 10.1016/j.ocarto.2023.100368. |
| Others, n = 8 | - Abbott H (2004): Classification-based physiotherapy is more effective than guidelines-based practice, for acute low back pain. In: NZ J PHYSIOTHER 32 (1) - Carey TS (1999): Chiropractic manipulation and McKenzie physiotherapy were not effective for low back pain…commentary on Cherkin DC, Deyo RA, Battié M et al. A comparison of physical therapy, chiropractic manipulation, and provision of an educational booklet for the treatment of patients with low back pain. N ENGL J MED 1998 Oct 8;339:1021-9. In: ACP J CLUB 130 (2), S. 42 - Haas, M. (2008): No difference in cost-effectiveness of intensive group training for chronic back pain compared with usual physiotherapy care. In: *Aust J Physiother* 54 (2), S. 144. DOI: 10.1016/s0004-9514(08)70051-5. |
|  | - Irrgang, J. J. (1997): Follow-up to the clinical and cost-effectiveness of two different programs for rehabilitation following ACL reconstruction. In: *J Orthop Sports Phys Ther* 26 (1), 39-40; author reply 40-6. DOI: 10.2519/jospt.1997.26.1.39. |
|  | - Nwachukwu, Benedict U. (2022): In Femoroacetabular Impingement Syndrome, Hip Arthroscopy Was Not Cost-Effective Compared with Personalized Hip Therapy at 1 Year. In: J BONE JOINT SURG (AM) 104 (22), S. 2036. DOI: 10.2106/JBJS.22.00964 - Snyder-Mackler, L. (1997): Follow-up to the clinical and cost-effectiveness of two different programs for rehabilitation following ACL reconstruction. In: *J Orthop Sports Phys Ther* 26 (1), 39; author reply 40-6. |
|  | - Stelzer JW, Agrawal R, Conaway W, Smith J, Martin SD. A Randomized Controlled Trial and Cost Analysis Assessing the Value of Physical Therapy for Treatment of Adhesive Capsulitis. Orthop J Sports Med. 2018 Jul 27;6(7 suppl4):2325967118S00173. doi: 10.1177/2325967118S00173 - Storheim, K. (2012): Targeted physiotherapy treatment for low back pain based on clinical risk can improve clinical and economic outcomes when compared with current best practice. In: *J Physiother* 58 (1), S. 57. DOI: 10.1016/s1836-9553(12)70073-5. |
| **Language, n = 3** |  |
|  | - Chen, C.; Li, D. W.; Wang, Q.; Xu, X. W.; Ma, Y. Z.; Li, Z.; Zou, W. L. (2016): [The cost effectiveness analysis of minimally invasive surgery and conservative treatment in elderly osteoporotic spinal fracture]. In: *Zhongguo Gu Shang* 29 (7), S. 614–618. DOI: 10.3969/j.issn.1003-0034.2016.07.006. |
|  | - Gialanella, B.; Prometti, P.; Ramponi, J. P.; Lazzarini, A.; Bonometti, G. (2013): [Rehabilitative and economic effectiveness of an outpatient rehabilition practice]. In: *G Ital Med Lav Ergon* 35 (2), S. 120–124. |
|  | - Navarrete-Peñaloza BI, Hernández-Amaro H. Costo médico directo de la rehabilitación tardía en trabajadores con fractura proximal de húmero de manejo conservador [Direct medical cost of late rehabilitation in workers with conservatively managed proximal humerus fracture]. Acta Ortop Mex. 2022 Jan-Feb;36(1):14-19. Spanish. PMID: 36099568 |
| **No access, n = 7** |  |
|  | - Abbott JH, Wilson R, Pinto D, The MOA Trial Team. Long-term cost-effectiveness of exercise therapy and/or manual therapy for hip or knee osteoarthritis: randomized controlled trial and computer simulation modelling. Osteoarthritis Cartilage 2019;27:S36. - AHC MEDIA (2020): Are Epidural Steroid Injections Cost-Effective? In: INTERN MED ALERT 42 (6), S. 1–2. Online verfügbar unter https://search.ebscohost.com/login.aspx?direct=true&db=cin20&AN=142690570&lang=de-de&site=ehost-live. - Alsayani, Khaled Yahya Abdullah; Baş Aslan, Ummuhan; Bayrak, Gökhan; Şavkın, Raziye; Büker, Nihal; Güngör, Harun Reşit (2023): Comparison of the effectiveness of late-phase clinic-based and home-based progressive resistance training in female patients with total knee arthroplasty. In: Physiother Theory Pract, S. 1–12. DOI: 10.1080/09593985.2023.2205925 |
| (cont.) No access, n = 7 | - Bakker C, Hidding A, Van Der Linden S, et al. Cost effectiveness of group physical therapy compared to individualized therapy for ankylosing spondylitis. A randomized controlled trial. J Rheumatol. 1994;21:264-268. - Brüggenjürgen, Bernd; Hamann, Bettina; Stukenborg-Colsman, Christina; Schulz, Friederike (2023): Ökonomische Evaluation der frühen Interdisziplinären Multimodalen Schmerztherapie allein und in Kombination mit der Ganzkörper-Elektrostimulation im Vergleich zur Standardversorgung bei Patienten mit chronischen nicht-spezifischen Rückenschmerzen. In: GESUNDHEITSOKONOMIE QUALITATSMANAGE 28 (6), S. 288–297. DOI: 10.1055/a-2042-2792 - Farid, M.; Ng, A. (2012): Cost analysis in the management of acute Achilles tendon rupture. In: MUSCLES LIGAMENTS TENDONS J, S. 39. Online verfügbar unter <https://search.ebscohost.com/login.aspx?direct=true&db=cin20&AN=87586062&lang=de-de&site=ehost-live>. - Saunders C; Hadler NM; Pearson JK; Shekelle PG (2001): Manual therapy for low back pain. In: PATIENT CARE 35 (10), S. 12–23. Online verfügbar unter https://search.ebscohost.com/login.aspx?direct=true&db=cin20&AN=107044164&lang=de-de&site=ehost-live. |
