# Supplementary material for: Cost-Effectiveness of Treatments for Musculoskeletal Conditions Offered by Physiotherapists: A Systematic Review of Trial-Based Evaluations
Source: Sports Med Open. 2024 Apr 13;10:38. doi: 10.1186/s40798-024-00713-9 (PMC11016054; doi:10.1186/s40798-024-00713-9)
Supplement: Supplementary file 3 — Additional file 3. Evaluation of the study quality with the Consensus on Health Economic Criteria checklist. [file 40798_2024_713_MOESM3_ESM.docx]

Table S3 Evaluation of the study quality with the Consensus on Health Economic Criteria checklist

| **Study** | **1** | **2** | **3** | **4** | **5** | **6** | **7** | **8** | **9** | **10** | **11** | **12** | **13** | **14** | **15** | **16** | **17** | **18** | **19** | **‘yes’- sum** |
| --- | --- | --- | --- | --- | --- | --- | --- | --- | --- | --- | --- | --- | --- | --- | --- | --- | --- | --- | --- | --- |
| Abbott et al., 2019 | yes | yes | yes | yes | yes | yes | yes | yes | yes | yes | yes | yes | yes | yes | yes | yes | yes | yes | yes | 19 |
| Aboagye et al., 2015 | yes | yes | yes | yes | yes | yes | no | yes | no | yes | yes | yes | yes | yes | yes | no | no | yes | yes | 15 |
| Ankjær-Jensen et al., 1994 | no | no | yes | yes | yes | yes | yes | yes | yes | yes | yes | yes | no | yes | yes | yes | no | no | no | 13 |
| Apeldoorn et al., 2012 | yes | yes | yes | yes | yes | yes | yes | yes | yes | yes | yes | yes | yes | yes | yes | yes | no | no | yes | 17 |
| Barker et al., 2019 | yes | yes | yes | yes | yes | yes | yes | yes | yes | yes | yes | yes | yes | yes | yes | yes | yes | yes | yes | 19 |
| Barker et al., 2020 | yes | yes | yes | yes | yes | yes | no | no | no | yes | yes | yes | yes | yes | yes | yes | no | yes | yes | 15 |
| Barnhoorn et al., 2018 | yes | yes+ | yes | no | yes | yes | yes | no | no | yes | yes | yes | no | yes | no | no | no | no | yes | 11 |
| Barton et al., 2009 | yes | yes | yes | yes | yes | yes | yes | yes | no | yes | yes | yes | yes | yes | yes | yes | yes | yes | yes | 18 |
| Bello et al., 2015 | yes | yes | yes | no | no | yes | no | no | no | yes | yes | yes | no | yes | no | yes | yes | yes | yes | 12 |
| Bennell et al., 2016 | yes | yes | yes | yes | yes | yes | no | yes | yes | yes | yes | yes | yes | yes | no | yes | yes | yes | yes | 17 |
| Bergman et al., 2010 | yes | yes | yes | yes | yes | yes | yes | yes | yes | yes | yes | yes | yes | yes | yes | yes | yes | yes | yes | 19 |
| Bosmans et al., 2011 | yes | yes | yes | yes | yes | yes | yes | yes | yes | yes | yes | yes | yes | yes | no | yes | yes | yes | yes | 18 |
| Bulthuis et al., 2008 | yes | yes | yes | yes | no | yes | yes | yes | yes | yes | yes | yes | yes | yes | no | no | no | no | no | 13 |
| Burton et al., 2004 | yes | yes | yes | yes | yes | yes | yes | yes | yes | yes | yes | yes | yes | yes | yes | yes | yes | yes | yes | 19 |
| Canaway et al., 2018 | yes | yes | yes | yes | yes | yes | yes | yes | yes | yes | yes | yes | yes | yes | yes | yes | yes | yes | yes | 19 |
| Carr et al., 2005 | yes | yes | yes | no | yes | yes | no | yes | yes | no | yes | yes | no | yes | no | yes | no | no | yes | 12 |
| Cherkin et al., 1998 | yes | yes | yes | no | yes | yes | yes | yes | yes | no | yes | no | no | no | no | yes | yes | no | no | 11 |
| Coombes et al., 2023 | yes | yes | yes | yes | yes | yes | yes | yes | yes | yes | yes | yes | yes | yes | yes | yes | yes | yes | yes | 19 |
| Coupé et al.‚ 2007 | yes | yes | yes | yes | yes | yes | yes | yes | yes | yes | yes | yes | yes | yes | yes | yes | no | yes | yes | 18 |
| Critchley et al., 2007 | yes | yes | yes | yes | yes | yes | yes | yes | yes | yes | yes | yes | yes | yes | yes | yes | yes | no | yes | 18 |
| Daker-White et al., 1999 | yes | no | yes | yes | yes | yes | yes | yes | yes | yes | yes | yes | no | yes | no | yes | no | yes | yes | 15 |
| Denninger et al., 2018 | yes | yes | yes | yes | yes | yes | yes | yes | yes | yes | yes | yes | no | no | no | yes | yes | yes | yes | 16 |
| Eggerding et al., 2021 | yes+ | yes+ | yes | yes | yes | yes | yes | yes | yes | yes | yes | yes | yes | yes | yes | yes | yes | yes | yes | 19 |
| Fernandes et al., 2017 | yes | yes | yes | yes | yes | yes | yes | yes | yes | yes | yes | yes | yes | yes | yes | yes | yes | yes | yes | 19 |
| Fernandez-de-las-penjas et al., 2019 | yes+ | yes | yes | yes | yes | yes | yes | yes | yes | yes | yes | yes | yes | yes | yes | yes | yes | yes | yes | 19 |
| Fritz et al., 2008 | yes | yes | yes | yes | yes | yes | yes | yes | yes | yes | yes | yes | no | yes | no | yes | no | no | no | 14 |
| Fritz et al., 2017 | yes | yes | yes | yes | yes | yes | yes | yes | yes | yes | yes | yes | yes | yes | yes | yes | no | yes | yes | 18 |
| Fusco et al., 2019 | yes | yes | yes | yes | yes | yes | yes | yes | yes | yes | yes | yes | yes | yes | yes | yes | yes | yes | no | 18 |
| Geraets et al., 2006 | no | yes | yes | yes | yes | yes | yes | yes | yes | yes | yes | yes | yes | yes | yes | yes | yes | no | yes | 17 |
| Griffin et al., 2022 | yes | yes | yes | yes | yes | yes | yes | yes | yes | yes | yes | yes | yes | yes | yes | yes | yes | yes | yes | 19 |
| Hahne et al., 2017 | yes+ | yes | yes | yes | yes | yes | yes | yes | yes | yes | yes | yes | yes | yes | yes | yes | yes | yes | yes | 19 |
| Heij et al., 2022 | yes | yes | yes | yes | yes | yes | yes | yes | yes | yes | yes | yes | yes | yes | yes | yes | yes | yes | yes | 19 |
| Herman et al., 2008 | yes | yes | yes | yes | yes | yes | yes | yes | yes | yes | yes | yes | yes | yes | yes | yes | yes | no | yes | 18 |
| Hlobil et al., 2007 | yes / | yes / | yes | yes | yes | yes | yes+ | yes | yes | yes | yes | yes | no | no | yes | yes | yes | no | no | 15 |
| Ho-Henriksson et al., 2022 | yes | yes | yes | yes | yes | yes | yes | yes | yes | yes | yes | yes | yes | yes | yes | yes | yes | yes | yes | 19 |
| Hopewell et al., 2021 | yes | yes | yes | yes | yes | yes | yes | yes | yes | yes | yes | yes | yes | yes | yes | yes | yes | yes | yes | 19 |
| Huang et al., 2012 | yes | yes | yes | yes | no | yes | no | yes | yes | yes | yes | yes | no | yes | no | yes | no | yes | yes | 14 |
| Hurley et al., 2007 | yes+ | yes | yes | yes | yes | yes | yes | yes | yes | yes | yes | yes | yes | yes | yes | yes | yes | yes | yes | 19 |
| Hurley et al., 2012 | yes+ | yes+ | yes | yes | yes | yes | yes | yes | yes | yes | yes | yes | no | yes | yes | yes | yes | no | yes | 17 |
| Hurley et al., 2015 | yes | yes | yes | yes | yes | yes | yes | yes | yes | yes | yes | yes | yes | yes | yes | yes | yes | yes | yes | 19 |
| James et al., 2005 | yes | yes | yes | yes | yes | yes | yes | yes | yes | yes | yes | yes | no | yes | yes | yes | yes | yes | yes | 18 |
| Jessep et al., 2009 | yes | yes | yes | yes | yes | yes | yes | yes | yes | yes | yes | yes | yes | yes | no | yes | yes | yes | yes | 18 |
| Johnson et al., 2007 | yes | yes | yes | yes | yes | yes | yes | yes | yes | yes | yes | yes | yes | yes | yes | yes | yes | yes | yes | 19 |
| Juhakoski et al., 2011 | yes | yes | yes | yes | yes | yes | yes | yes | yes | yes | yes | yes | no | no | no | yes | yes | yes | yes | 16 |
| Karjalainen et al., 2003 | yes | yes | yes | yes | yes | yes | yes | yes | yes | yes | yes | yes | no | yes | no | yes | yes | yes | yes | 17 |
| Kigozi et al., 2018 | yes | yes | yes | yes | yes | yes | yes | yes | yes | yes | yes | yes | yes | yes | yes | yes | yes | yes | yes | 19 |
| Kim et al., 2020 | yes | yes | yes | yes | no | yes | yes | yes | yes | yes | yes | yes | no | yes | no | yes | yes | yes | yes | 16 |
| Knoop et al., 2023 | yes | yes | yes | yes | yes | yes | yes | yes | yes | yes | yes | yes | yes | yes | yes | yes | yes | yes | yes | 19 |
| Korthals-de Bos et al., 2003 | yes | yes | yes | yes | yes | yes | yes | yes | yes | yes | yes | yes | yes | yes | yes | yes | yes | yes | yes | 19 |
| Korthals-de Bos et al., 2004 | / | yes | yes | yes | yes | yes | yes | yes | yes | yes | yes | yes | yes | yes | no | yes | yes | yes | no | 16 |
| Leininger et al., 2016 | yes | yes | yes | yes | yes | yes | yes | yes | yes | yes | yes | yes | yes | yes | yes | yes | yes | no | no | 17 |
| Lewis et al., 2007 | yes+ | yes | yes | yes | yes | yes | yes | yes | yes | yes | yes | yes | yes | yes | yes | yes | yes | yes | yes | 19 |
| Lilje et al., 2014 | yes+ | yes | yes | yes | yes | yes | yes | yes | yes | yes | yes | yes | no | yes | yes | yes | yes | yes | yes | 18 |
| Lin et al., 2008 | yes | yes | yes | yes | yes | yes | yes | yes | yes | yes | yes | yes | no | yes | no | yes | yes | yes | yes | 17 |
| Manca et al., 2006 | yes+ | yes | yes | yes | yes | yes | yes | yes | yes | yes | yes | yes | yes | yes | yes | yes | yes | no | yes | 18 |
| Manca et al., 2007 | yes+ | yes | yes | yes | yes | yes | yes | yes | yes | yes | yes | yes | yes | yes | yes | yes | yes | yes | yes | 19 |
| McCarthy et al., 2004 | yes | yes | yes | yes | yes | yes | yes | yes | yes | yes | yes | yes | yes | yes | yes | yes | yes | yes | yes | 19 |
| Mitchell et al., 2005 | yes | yes | yes | yes | yes | yes | yes | yes | yes | yes | yes | yes | no | yes | yes | yes | yes | no | yes | 17 |
| Müller et al., 2019 | yes | yes | yes | yes | yes | yes | yes | yes | yes | yes | yes | yes | no | no | no | yes | yes | yes | yes | 16 |
| Niemistö et al., 2005 | yes+ | yes | yes | yes | yes | yes | yes | yes | yes | yes | yes | yes | yes | yes | no | yes | no | yes | no | 18 |
| Niemistö et al., 2003 | yes | yes | yes | yes | yes | yes | yes | yes | yes | yes | yes | yes | yes | yes | no | yes | yes | yes | yes | 16 |
| Pinto et al., 2013 | yes | yes+ | yes | yes | yes | yes | yes | yes | yes | yes | yes | yes | yes | yes | yes | yes | yes | yes | yes+ | 19 |
| Pryymachenko et al., 2021 | yes | yes | yes | yes | yes | yes | yes | yes | yes | yes | yes | yes | yes | yes | yes | yes | yes | yes | yes | 19 |
| Rhon et al., 2022 | yes | yes | yes | yes | yes | yes | yes | yes | yes | yes | yes | yes | yes | yes | yes | yes | yes | yes | yes | 19 |
| Rivero-Arias et al., 2006 | yes | yes | yes | yes | yes | yes | yes | yes | yes | yes | yes | yes | yes | yes | yes | yes | no | yes | no | 17 |
| Sevick et al., 2000 (ex) | yes | yes | yes | yes | yes | yes | yes | yes | yes | yes | yes | yes | yes | no | no | yes | yes | no | yes | 17 |
| Sevick et al., 2000 (life) | yes+ | yes | yes | yes | yes | yes | yes | yes | yes | yes | yes | yes | yes | yes | yes | yes | yes | no | yes | 16 |
| Sevick et al., 2009 | yes | yes | yes | yes | yes | yes | yes | yes | yes | yes | yes | yes | no | yes | yes | yes | yes | no | yes | 18 |
| Skargren et al., 1997 | yes | yes | yes | yes | yes | yes | yes | yes | yes | yes | yes | yes | no | yes | no | yes | yes | no | yes | 16 |
| Skargren et al., 1998 | yes | yes+ | yes | yes | yes | yes | yes | yes | yes | yes | yes | yes | no | yes | yes | yes | yes | no | no | 16 |
| Smeets et al., 2009 | yes | yes | yes | yes | yes | yes | yes | yes | yes | yes | yes | yes | yes | no | yes | yes | yes | no | yes | 17 |
| Søgaard et al., 2008 | yes | yes | yes | yes | yes | yes | yes | yes | yes | yes | yes | yes | yes | yes | yes | yes | yes | no | yes | 18 |
| Stan et al., 2015 | yes | yes | yes | yes? | no | yes | no | yes | yes | yes | yes | yes | yes | yes | no | yes | no | yes | yes | 15 |
| Struijs et al., 2006 | yes | yes | yes | yes | yes | yes | yes | yes | yes | yes | yes | yes | yes | yes | yes | yes | no | yes | yes | 18 |
| Suni et al., 2018 | yes | yes | yes | yes | yes | yes | yes | yes | yes | yes | yes | yes | yes | yes | yes | yes | yes | yes | yes | 19 |
| Tan et al., 2010 | yes | yes | yes | yes | yes | yes | yes | yes | yes | yes | yes | yes | yes | yes | no | yes | yes | no | yes | 17 |
| Tan et al., 2016 | yes | yes | yes | yes | yes | yes | yes | yes | yes | yes | yes | yes | yes | yes | yes | yes | yes | yes | yes | 19 |
| van de Graaf et al., 2020 | yes | yes | yes | yes | yes | yes | yes | yes | yes | yes | yes | yes | yes | yes | yes | yes | yes | yes | yes | 19 |
| van der Graaff et al., 2023 | yes | yes | yes | yes | yes | yes | yes | yes | yes | yes | yes | yes | yes | yes | yes | yes | yes | yes | yes | 19 |
| Van den Hout et al., 2005 | yes | yes | yes | yes | yes | yes | yes | yes | yes | yes | yes | yes | no | no | yes | yes | yes | no | yes | 16 |
| van der Roer et al., 2008 | no | yes+ | yes | yes | yes | yes | yes | yes | yes | yes | yes | yes | yes | yes | yes | yes | yes | yes | yes | 18 |
| Van Dongen et al., 2016 | yes | yes | yes | yes | yes | yes | yes | yes | yes | yes | yes | yes | yes | yes | yes | yes | yes | yes | yes | 19 |
| Whitehurst et al., 2007 | yes+ | yes | yes | yes | yes | yes | yes | yes | yes | yes | yes | yes | yes | yes | yes | yes | yes | no | no | 17 |
| 1 Is the study population clearly described? 2 Are competing alternatives clearly described? 3 Is a well-defined research question posed in answerable form? 4 Is the economic study design appropriate to the stated objective? 5 Is the chosen time horizon appropriate to include relevant costs and consequences? 6 Is the actual perspective chosen appropriate? 7 Are all important and relevant costs for each alternative identified? 8 Are all costs measured appropriately in physical units? 9 Are costs valued appropriately? 10 Are all important and relevant outcomes for each alternative identified? 11 Are all outcomes measured appropriately? 12 Are outcomes valued appropriately? 13 Is an incremental analysis of costs and outcomes of alternatives performed? 14 Are all future costs and outcomes discounted appropriately? 15 Are all important variables, whose values are uncertain appropriately subjected to sensitivity analysis? 16 Do the conclusion follow the data reported? 17 Does the study discuss the generalizability of the results to other settings and patient/client groups? 18 Does the article indicate that there is no potential conflict of interest of study researcher(s) and funder(s)? 19 Are ethical and distributional issues discussed appropriately? | | | | | | | | | | | | | | | | | | | | |
| / reference to another paper, which is not accessible; + information found in an additional paper | | | | | | | | | | | | | | | | | | | | |
